# Handling of Missing Outcome Data in Traumatic Brain Injury Research: A Systematic Review

**DOI:** 10.1089/neu.2018.6216

**Published:** 2019-09-10

**Authors:** Sophie Richter, Susan Stevenson, Tom Newman, Lindsay Wilson, David K. Menon, Andrew I.R. Maas, Daan Nieboer, Hester Lingsma, Ewout W. Steyerberg, Virginia F.J. Newcombe

**Affiliations:** ^1^ University Division of Anaesthesia, Department of Medicine, University of Cambridge, Cambridge, United Kingdom.; ^2^ Division of Psychology, University of Stirling, Stirling, United Kingdom.; ^3^ Department of Neurosurgery, Antwerp University Hospital and University of Antwerp, Edegem, Belgium.; ^4^ Department of Public Health, Erasmus MC University Medical Center Rotterdam, Rotterdam, the Netherlands.; ^5^ Department of Biomedical Data Sciences, Leiden University Medical Center, Leiden, the Netherlands.

**Keywords:** follow-up, missing data, multiple imputation, traumatic brain injury

## Abstract

Traumatic brain injury (TBI) research commonly measures long-term functional outcome, but studies often suffer from missing data as patients are lost to follow-up. This review assesses the extent and handling of missing outcome data in the TBI literature and provides a practical guide for future research. Relevant electronic databases were searched from January 1, 2012 to October 27, 2017 for TBI studies that used the Glasgow Outcome Scale or Glasgow Outcome Scale-Extended (GOS/GOSE) as an outcome measure. Studies were screened and data extracted in line with Cochrane guidance.

A total of 195 studies, 21 interventional, 174 observational, with 104,688 patients were included. Using the reported follow-up rates in a mixed model, on average 91% of patients were predicted to return to follow-up at 6 months post-injury, 84% at 1 year, and 69% at 2 years. However, 36% of studies provided insufficient information to determine the number of subjects at each time-point. Of 139 studies that did report missing outcome data, only 50% attempted to identify why data were missing, with just 4 reporting their assumption on the “missingness mechanism.” The handling of missing data was heterogeneous, with the most common method being its exclusion from analysis. These results confirm substantial variability in the standard of reporting and handling of missing outcome data in TBI research. We conclude that practical guidance is needed to facilitate meaningful and accurate study interpretation, and therefore propose a framework for the handling of missing outcome data in future TBI research.

## Introduction

Traumatic brain injury (TBI) studies commonly suffer from patient attrition resulting in missing outcome data.^1–3^ Inadequate handling of missing data can decrease power, violate intention-to-treat analyses, and introduce bias. Missing data first gained attention in the 1970s when Rubin introduced the concept of the “missingness mechanism” or the reason why data are missing.^4^ The missingness mechanism describes data as “missing completely at random” (MCAR), “missing at random” (MAR), or “missing not at random” (MNAR). Importantly, the mechanism is not only relevant to the interpretation of conclusions, but also informs what statistical approaches may best be used for handling missing data.

In MCAR, patients missing due to loss to follow-up do not differ in any respect from those remaining, leaving conclusions unaffected. However, in most clinical research, MCAR rarely applies with previous studies in TBI research assuming missing data to be MAR.^1–3^ MAR occurs when loss to follow-up is random, conditional on observed and measurable patient characteristics and can therefore be statistically corrected for. In MNAR however, the outcome itself (or unobserved characteristics related to missingness) determines likelihood of follow-up. Its identification requires knowledge of the population, disease, and follow-up process as no statistical test alone is able to identify data as MNAR. Importantly, given that this is unmeasurable, the bias introduced by MNAR cannot be overcome by statistical correction alone and requires additional techniques. Therefore, researchers should apply caution in drawing conclusions when lost patients differ from those remaining in the study due to the potential of missing data being MNAR.

The hazard of missing data to accurate study interpretation has been recognized by multiple regulatory bodies including the European Medicine Agency (EMA), National Research Council, and Food and Drug Administration.^5–7^ The EMA provides specific recommendations for handling missing values in clinical trials, where unbalanced attrition between treatment arms can introduce bias. Recommendations include study design features to minimize the amount of missing data, anticipation of missing data with predefined plans for analyses, and exploration of the missingness mechanism with post hoc sensitivity analyses. Although similar principles can be applied to observational studies, specific guidelines relating to handling of missing data in observational studies are lacking. Given the predominance of observational studies in TBI research including emerging, large volume data series, guidance is urgently required.

The aims of this review are therefore two-fold. First, to assess the quantity, reporting, and handling of missing outcome data in longitudinal studies of TBI, including both clinical trials and observational studies. Second, to provide an explicit guide for TBI researchers on how to report and handle their missing outcome data.

## Methods

This review was conducted and reported in line with the Cochrane guidelines for methodological reviews and the Preferred Reporting Items for Systematic reviews and Meta-Analyses (PRISMA) statement for systematic reviews.^8,9^ The protocol for this systematic review was registered on PROSPERO and is accessible online (www.crd.york.ac.uk/PROSPERO/display_record.php?ID=CRD42017080788).^10^

### Information sources

The following databases were searched up to October 27, 2017: MEDLINE via OVID, Embase via OVID, Cochrane Central Register of Controlled Trials, and Cumulative Index of Nursing and Allied Health Literature (CINAHL). Search terms relating to traumatic brain injury were taken from a previously published living review by the CENTER-TBI group^11^ and included exploded MESH terms for “Brain Injuries” or “Craniocerebral Trauma” or word variations of “((head* or brain*) adj2 (injur* or trauma*))” in the title, abstract, or keywords.^12^ These were combined with the following search terms relating to the Glasgow Outcome Scale (GOS): the exploded MESH term for “Glasgow Outcome Scale”; or word variations of “(Glasgow Outcome Scale or extended Glasgow Outcome Scale or GOS or GOSE)” or “((functional or neur*) adj (status or outcome*))” in the title, abstract, or key words. The search was restricted to studies on human participants. As missing data guidelines for interventional studies were published in 2010,^5–7^ we only included studies published after 2011 to allow authors sufficient time to familiarize themselves with the guidelines and apply them to their study analyses.

### Inclusion and exclusion criteria

We included traumatic brain injury studies where GOS^13^ or Glasgow Outcome Scale-Extended (GOSE)^14^ was used as a longitudinal outcome measure. Although a variety of outcome measures are employed in TBI research, we were concerned that some outcome measures could be more prone to non-response than others. To reduce heterogeneity and allow for a valid comparison of missing data among the studies, we opted to only select studies with a common outcome measure. GOS/GOSE was chosen as it is the most widely used outcome measure and would thus generate the most representative sample of TBI studies.

GOS was one of the first outcome scores to assess functional outcome following TBI and remains the most widely cited and validated score in TBI. The extended version (which includes an 8-point scale rather than a 5-point scale) was also included, with the two versions showing similar validity.^15^ GOS is currently recommended in head injury by both the National Institute of Neurological Disorders and Stroke in the United States^16^ and the Department of Health in the United Kingdom.^17^ For the same reasons we only accepted studies using the adult version of GOS/GOSE. A modified version of GOSE Peds that takes account of developmental stage exists and shows a strong association with the standard version.^18^ Therefore, we accepted studies with a small proportion of children (<25% of patients under 16 years of age) and excluded studies that focused on pediatric TBI specifically. Studies that included non-traumatic etiologies of brain injury were also excluded.

Longitudinal was defined as any study that recruited patients or started data collection in the acute or subacute phase (within 2 months of injury) and had a single time-point or multiple follow-up time-points at least 3 months after injury. We selected larger TBI studies with an arbitrary minimum of 100 patients, in only the English language as this language was shared by all reviewers.

Both interventional and observational TBI studies were included. Interventional studies were defined as involving allocation of an intervention (investigative or therapeutic) by the researcher to a study group with a comparison arm. Interventional studies included both randomized and non-randomized clinical trials (RCTs and non-RCTs) differing by the use of randomization in the process of allocation. In non-randomized clinical trials, treatment or control allocation occurs through an alternative process from randomization. In terms of observational studies, before-and-after studies and cohort studies were included. Cohorts were defined as studies observing the effect of a natural exposure on outcome using an unexposed group as a control. Both retrospective and prospective observational studies were included. Before-and-after studies were defined as studies where interventions arose naturally or unintentionally without formal assignment or allocation with the treatment effect being compared before and after its implementation.

Case-control studies, case reports, and case series were excluded, as by definition, these study designs do not have missing outcome data. Incomplete publications such as posters, abstracts, and synopses were also excluded as detailed reporting was required for data extraction.

### Study selection and data extraction

Study selection was done using the Covidence systematic review software.^19^ Initial screening of abstracts and titles by one reviewer (SS or SR) excluded irrelevant articles. Eligibility of full texts was assessed by two reviewers independently (two of SR, SS, and TN).

Data extraction from full texts was performed in duplicate by two independent reviewers (two of SS, SR, and TN), using a piloted extraction form. Information was extracted on study design (interventional or observational), cohort characteristics (age and severity of TBI in terms of Glasgow Coma Scale [GCS]) and features of follow-up. Specifically, we looked at the version of GOS used, its use as a primary or secondary outcome, and the timing, frequency, and success of follow-up, defined as the proportion of missing patients. Timing of follow-up was recorded as occurring at discrete time-points or within a range of months from initial injury. Pooling GOS data across a time range is a methodological approach that itself reduces missing outcome data and was analyzed separately. When loss to follow-up was not explicitly reported in the text body, the numbers of patients missing was calculated using values quoted in the figures and tables when available. Patients who died prior to follow-up were not considered to be missing, as their outcome was known (GOS 1).

We identified and classified five patterns of reporting of missing outcome data:

1. Sufficient. Authors report complete follow-up achieved on all patients.
2. Sufficient. Patients lost to follow-up. Authors clearly state number of patients lost at each time-point.
3. Insufficient. Patients lost to follow-up and total missing reported, but timing unclear.
4. Insufficient. Patients lost to follow-up but number missing unclear.
5. Insufficient. Authors do not report if patients were missing.

Patterns 1 and 2 are described as sufficient as it is transparent how many patients were followed up or missing. Studies using pattern 1 explicitly state they have no missing outcome data. Similarly, pattern 2 studies provide sufficient information to deduce how many patients were missing at each follow-up point. The remaining three patterns reflect insufficient reporting. In pattern 3, authors follow up patients at more than one time-point. Although they report how many patients were lost overall, it is unclear at which time-point patients drop out. In pattern 4, authors state patients are lost to follow-up but not how many. Studies using pattern 5 offer no comment on the follow-up rate, nor do they provide information in figures or tables on whether patients were lost. Therefore, it is unclear whether they indeed managed to follow up all patients or failed to declare their missing data.

In addition to identifying the reporting pattern, we described studies in reference to populations they included. Studies that recorded the baseline characteristics of all patients initially enrolled, irrespective of successful follow-up were labeled as having an “inclusive approach.” Alternatively, studies that described only patients successfully followed up were considered as having an “exclusive approach.”

For studies with missing outcome data, we recorded whether authors explored or stated explicitly the assumed missingness mechanism. We considered there to be two ways of exploring missingness. First, authors could compare baseline characteristics of retained with missing patients, or alternatively, they could compare the proportion of missing data in interventional or non-interventional arms in the case of interventional studies or exposed and unexposed patients in observational studies.

In the event of missing data, we recorded the choice of handling technique and the use of a sensitivity analyses. Techniques for handling missing data included omission, imputation, and other advanced statistical analyses. Omission, also known as listwise deletion involves omission of missing outcome data from the analysis. Imputation refers to the substitution of the missing value with an estimate. This can be single or multiple, depending on whether the data gap is filled by one or several plausible estimates. Advanced statistical techniques use all available information in the data set to predict outcome. An example includes expectation-maximization algorithms as used in multi-level modeling. We considered the techniques in reference to the number of time-points, either single or multiple, as this has implications on the appropriate technique for use.

Once data extraction was complete, two reviewers (SS and SR) searched for duplicate publications on the same patient cohorts to ensure a cohort would be included only once. This was done by comparing numbers, dates, centers, and inclusion criteria for recruitment across all texts. We considered a text to be a duplicate if another article reported on the identical cohort or reported on a larger cohort that fully included the smaller cohort. For inclusion in this review we chose the text that included the larger patient cohort, or in case of identical size, was published first. If patient cohorts overlapped only partially, we treated each as a unique cohort. Disagreements at any stage were resolved through discussion and consensus with a third reviewer.

### Assessment of risk of bias

To assess the quality of research being reviewed, we performed an assessment of risk of bias. This was done in accordance with the Cochrane Handbook for Systematic Reviews of Interventions^8^ using the Cochrane risk of bias tool for clinical trials^20^ and the Newcastle-Ottawa Scale^21^ for cohort studies (see Supplementary Appendix S1 for details). Briefly, the Cochrane risk of bias tool looks at seven areas in trial design and implementation with potential for the introduction of bias. The risk is assessed as high, low, or unclear. Similarly, the Newcastle-Ottawa Scale assesses the quality of non-randomized studies including cohorts, in eight domains using a star system. Each domain could score “1 star” depending on quality, with the exception of “comparability of cohorts” where 2 stars could be awarded when multiple confounders were controlled for. Two reviewers assessed risk of bias independently for each study (two of SS, SR, and TN). Disagreements were resolved through discussion and consensus with the third reviewer.

### Data synthesis

Data were only synthesized for studies that fulfilled eligibility criteria at full-text review and were included after removing duplicate studies on the same patient cohort.

Quantification of missing data was done in two steps, depending on whether studies used an inclusive or exclusive approach. The first step was to calculate how many patients were initially enrolled in each study. The second step involved calculating the number of patients whose outcome was missing, that is, patients who were recruited to the study but lacked an outcome.

Authors of studies using the inclusive approach reported the baseline data on the number of patients enrolled in their study, irrespective of follow-up. Subsequently, they described the number of patients at each follow-up time-point. This could be either the same number in the case of complete follow-up, or a reduced number due to dropout. The percentage of patients missing was calculated as per Equation 1:

**Figure:**
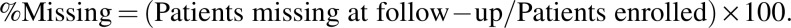


Studies with the exclusive approach, however, provided baseline data only on the number of patients successfully followed up. If they also described the number of patients excluded because of lack of follow-up, this number was added to the number of patients described and the percentage of missing data calculated as per Equation 2:

**Figure:**
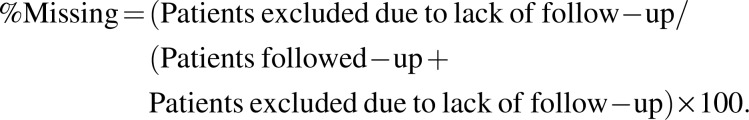


If studies only reported how many patients were successfully followed up but did not say how many patients were excluded due to loss to follow-up, then the original number of patients enrolled in the study could not be calculated.

If studies using the exclusive approach had only contacted patients at a single time-point, then we knew that all loss to follow-up had occurred at that time-point. If studies excluded patients due to loss to follow-up and had tried to contact patients at multiple time-points, the first of which was hospital discharge, we assumed that loss to follow-up had occurred at the second time-point.

### Statistical analysis

Data were analysed in R version 3.5.0.^22^ Fisher's exact test was used to compare categorical variables and the Mann-Whitney U test was used for continuous variables. We compared values between interventional and observational studies and stated any statistically significant results. In total, seven variables were compared between observational and interventional studies, and again between RCTs and non-RCTs, yielding 14 comparisons. For more robust conclusions, a *p*-value threshold of 0.005 was chosen.^23^

Fifteen (7.7%) of the 195 studies did not report on the quantity of missing outcome. When comparing these with the 180 studies that did report the proportion of patients missing, no difference was found in the proportion of interventional versus observational studies (*p* = 1.00), retrospective versus prospective studies (*p* = 1.00), studies involving pediatric patients (*p* = 0.781), studies using GOS as a primary versus secondary outcome (*p* = 1.00), the severity profile of patients (*p* = 1.00), and the study size as judged by the number of patients successfully followed up (*p* = 0.677). Formal comparison of these studies with and without reporting, using Little's MCAR test^24^ (from R package “BaylorEdPsych” version 0.5), showed that the MCAR assumption was not rejected (χ^2^ 4.6, *p* = 0.47). Thus, we simply omitted the 15 studies from graphical representations relating to the percentage of missing data.

Multi-level modeling of follow-up rates was performed in R using package “lme4” version 1.1–18-1, with “Time” as a fixed effect. Studies that pooled GOS across a time range rather than discrete time-points, and studies that did not report the follow-up rate were excluded. Thus, a total of 156 studies with 368 follow-up time-points were included. To account for within-study correlation of follow-up rates over time, “Study ID” was used as a random effect to allow for study specific intercepts and slopes.

## Results

### Search results

**FIG. 1.**
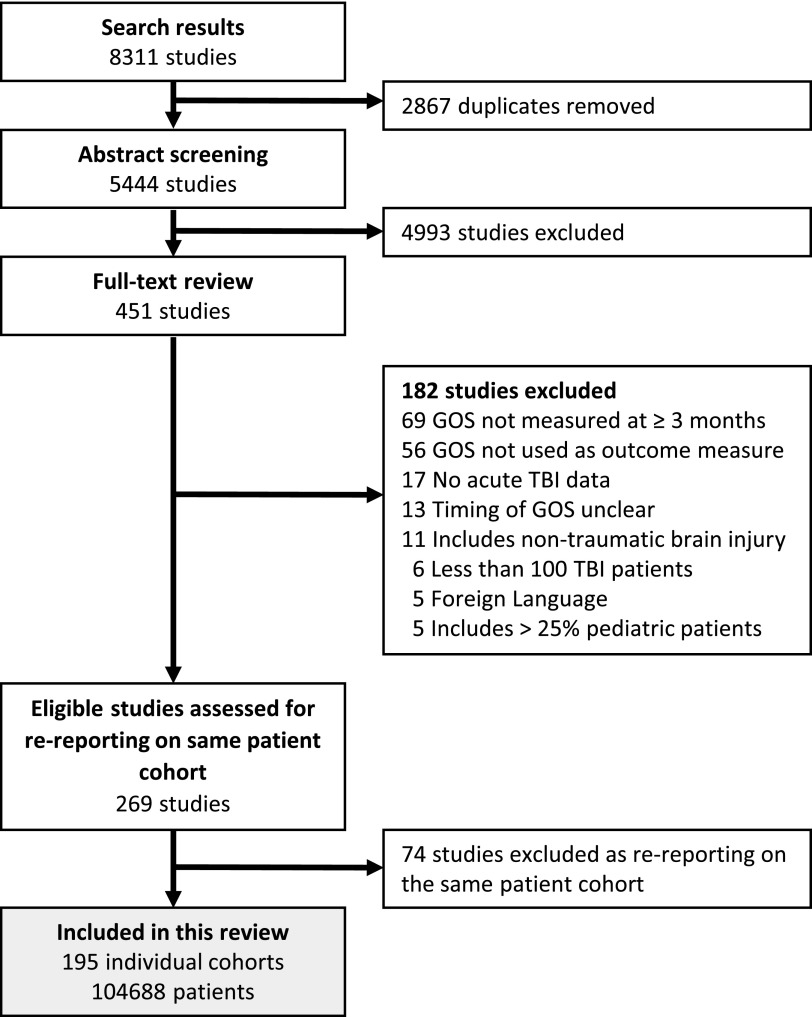
PRISMA flowchart illustrating the search and study inclusion process.

The search yielded 269 eligible articles, which reported on 195 unique patient cohorts (Fig. 1). The characteristics of these 195 cohorts, henceforth called “studies,” are summarized in Table 1 (for more detail see Supplementary Appendix S2). Overall the risk of bias in these studies was judged to be low to moderate (Supplementary Appendix S1).

**Table 1.** Study Characteristics of Included Studies

*Study characteristics*		*Interventional*	*Observational*	*Total*
Total	Studies	21	174	195
	Number of patients enrolled	7774	96,914	104,688^*^
Number of studies by injury severity	All severities	5	73	78
	Mild	0	8	8
	Moderate and severe	16	90	106
	Not reported	0	3	3
Studies including pediatric patients		6 (29%)	46 (26%)	52 (27%)
GOS/GOSE is the primary outcome		20 (95%)	143 (82%)	163 (84%)
Follow-up at non-discrete time-points Number (% of studies)		1 (5%)	24 (14%)	25 (13%)
Number of follow-up time-points Mode (range)		1 (1-2)	1 (1-5)	1 (1-5)
Studies with multiple follow-up time-points Number (% of studies)		6 (21%)	43 (25%)	49 (25%)
Follow up duration in months Mean (range)		7.7 (3-26)	12.2 (3-120)	11.6 (3-120)

^*^ Number for the 180 studies for which the number of patients enrolled could be discerned.

GOS/GOSE, Glasgow Outcome Scale/Glasgow Outcome Scale-Extended.

Twenty-one studies were interventional in design compared with 174 observational studies. Of the 21 interventional studies, there were 17 parallel RCTs, one cluster RCT, and 3 non-RCTs. Only 1 of the 174 observational studies was a before-and-after study; the remaining 173 were cohort studies.

### Reporting of missing outcome data

Reporting of follow-up was considered sufficient in 64% of studies. In 14% of studies with sufficient reporting, missing outcome data were deduced through analysis of figures and tables rather than explicit description in the text. Although most studies reported sufficiently, 23% did not report anything on missing outcomes (Table 2).

**Table 2.** Reporting of Follow-Up Utilized Five Patterns

*Reporting of follow-up*	*Studies*	*Studies with explicit reporting*	*Patients lost to follow-up*
1. Sufficient: Report achieving complete follow-up	10 (5%)	10 (100%)	0 (0%)
2. Sufficient: Report number of patients missing at each time-point	115 (59%)	98 (85%)	18,324 (22%)
3. Insufficient: Report number of patients missing, but timing unclear	9 (5%)	6 (75%)	1559 (20%)
4. Insufficient: Report patients were missing, but number unclear	15 (8%)	15 (100%)	Not reported
5. Insufficient: Do not report if patients were missing	46 (23%)	0 (0%)	Presumed zero
Total	195 (100%)	129 (66%)	>19,883 (>19%)

In patterns 1, 2, and 3, authors provide enough information to deduce the total number of patients lost to follow up. In patterns 4 and 5 it is not reported or presumed to be zero. Therefore, the total number of patients missing in all the studies is unknown and a minimum estimate is given in the bottom right column. “Studies with explicit reporting” describes studies that state clearly in the text if patients were missing and how many.

### Amount of missing outcome data

**FIG. 2.**
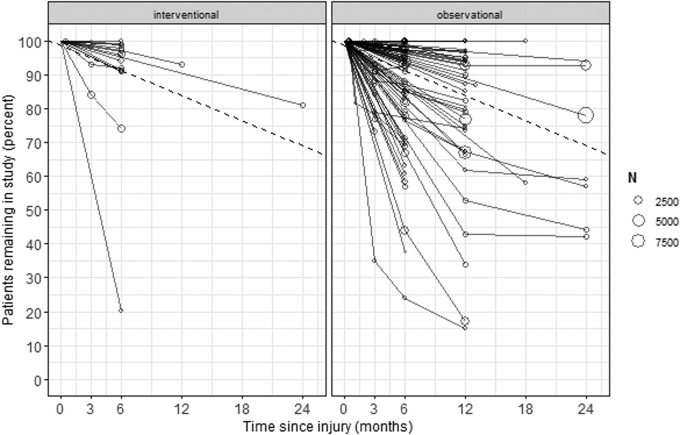
Patient follow-up rate in TBI studies. The left-hand panel displays interventional studies, the right-hand panel observational studies. Each line represents one study; the circles mark the follow-up time-points with the size reflecting the number of patients initially enrolled. The dashed line indicates the mean follow-up rate as predicted from the multi-level model. TBI, traumatic brain injury.

Follow-up rates varied widely across studies (Fig. 2) and could only be calculated when both the number enrolled and the number missing at a specific time-point were known. This occurred in 156 studies. Follow-up rates could not be extracted in 39 studies where the number of patients initially enrolled could not be calculated (14 studies), GOS was assessed across a range (24 studies), or where both situations applied (1 study).

Multi-level modeling predicts a follow-up rate of 91% at 6 months, dropping to 84% at 12 months and 69% at 24 months for both interventional and observational studies. There was no significant difference in the follow-up rate between interventional and observational studies (*p* = 0.528); however, we infrequently observed follow-up beyond 6 months in interventional studies.

In 25 (13%) studies, time to follow-up was presented as a range and varied considerably within the study. GOS values were pooled with ranges varying from 3 months to 54 years. The amount of missing outcome data present in range studies (mean 21%, median 15%) compared with studies with discrete time-points (mean 12%, median 5%) represented a non-significant trend toward better follow-up in studies with discrete time-points (*p* = 0.039).

### Approaches to missing outcome data

**FIG. 3.**
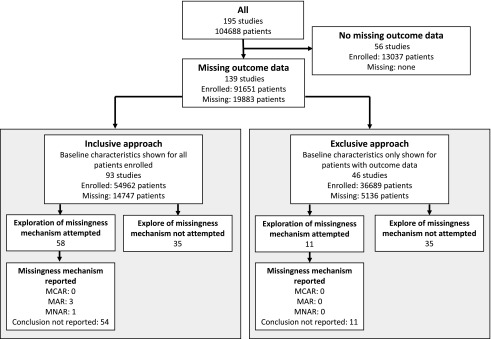
Approaches to handling missing data. Nearly half of all studies attempted to explore the “missingness mechanism.” However, only 4 of all studies explicitly stated the missingness mechanism, with 65 of 69 studies not mentioning a mechanism. *For 15 studies it is unclear how many patients were enrolled prior to loss to follow-up, so these patients are excluded from the numbers in this flowchart.

In studies with missing outcome data, we observed two broad approaches to the handling of missing data, referred to as “inclusive” and “exclusive” approaches (Fig. 3).

The inclusive approach was used by two thirds of studies (93 of 139 studies) with missing outcome data (Fig. 3) and the exclusive approach was used by the remaining third (46 of 139 studies; Fig. 3). There was a trend of interventional studies preferring the inclusive approach compared with observational studies (89% vs. 64%, *p* = 0.035).

### Missingness mechanism

An exploration of the missingness mechanisms was attempted by 69 (50%) of all 139 studies with missing data (Fig. 3). This was done either by comparing baseline characteristics between retained and lost patients (28 studies, 20%) and/or by comparing attrition rates across patient groups (50 studies, 36%). A comparison of retained and lost patients took the form of a description in text (3 studies), descriptive statistics (7 studies), and statistical testing (18 studies). The most common covariates compared included age (86% of comparisons), TBI severity (75%), sex (71%), mechanism of injury (39%), and imaging findings (35%). Additional features compared infrequently included ethnicity, pre-morbid education and employment status, alcohol abuse, and need for surgery. Of these studies, 16 (57%) found a difference between the patients lost and those followed up.

Attrition rates were compared between study arms in 17 of the 18 interventional studies with missing data, with two finding a substantial difference. Conversely, only 33 of 121 observational studies with missing outcome data compared attrition rates according to the exposure of interest with 10 finding a significant difference. Thus, observational studies were less likely than trials to compare attrition rates across groups (*p* < 0.0001).

Although 50% of studies appeared to make some attempt to identify the missingness mechanism, only four explicitly stated which missingness mechanism they decided to assume for their analysis: three MAR, one MNAR (Fig. 3).

### Handling missing values

Techniques used for handling missing data varied considerably across studies (Table 3). There was no relationship between the choice of technique and amount of missing outcome data.

**Table 3.** Frequency of Handling Techniques Used in the 139 Studies with Missing Outcome Data

*Handling technique*	*Follow-up time-points*	
	*Single time-point, number of studies (percent)*	*Multiple time-points, number of studies (percent)*
Exclusive approach	42 (43%)	4 (10%)
Omission	43 (44%)	33 (79%)
Omission plus single imputation^*^	4 (4%)	0
Single imputation	3 (3%)	3 (7%)
Multiple imputation	3 (3%)	0
Multi-level model	1 (1%)	2 (5%)
Analysis abandoned	1 (1%)	0
Total	97 (100%)	42 (100%)

^*^ In all cases, omission was combined with “last observation carried forward” as the technique of single imputation.

A sensitivity analysis was performed by only 5 of 139 studies with missing data. All 5 compared single or multiple imputation to omission.

## Discussion

We systematically reviewed the reporting and handling of missing outcome data in 195 TBI studies with more than 100,000 patients. Attaining complete follow-up for all patients in a study is extremely challenging and was only achieved for certain in 5% of studies in our data set. Although we acknowledge this difficulty, accurate reporting of the number of patients enrolled and subsequently followed up is simple and allows readers to meaningfully interpret outcome data. However, we found over a third of studies did not provide sufficient information for the reader to discern how many patients had contributed to the outcome data at defined time-points.

The first step in deciding how to deal with missing values is to explore why these data are missing, identify the missingness mechanism, and whether there is a systematic difference between patients with and without available outcome data. Only half of the studies attempted this and only four studies documented which missingness mechanism they assumed. This is surprising considering that the missingness mechanism determines how missing data should be handled, and suggests reliance on the default in statistical programs, which is usually to exclude patients without available data.

**FIG. 4.**
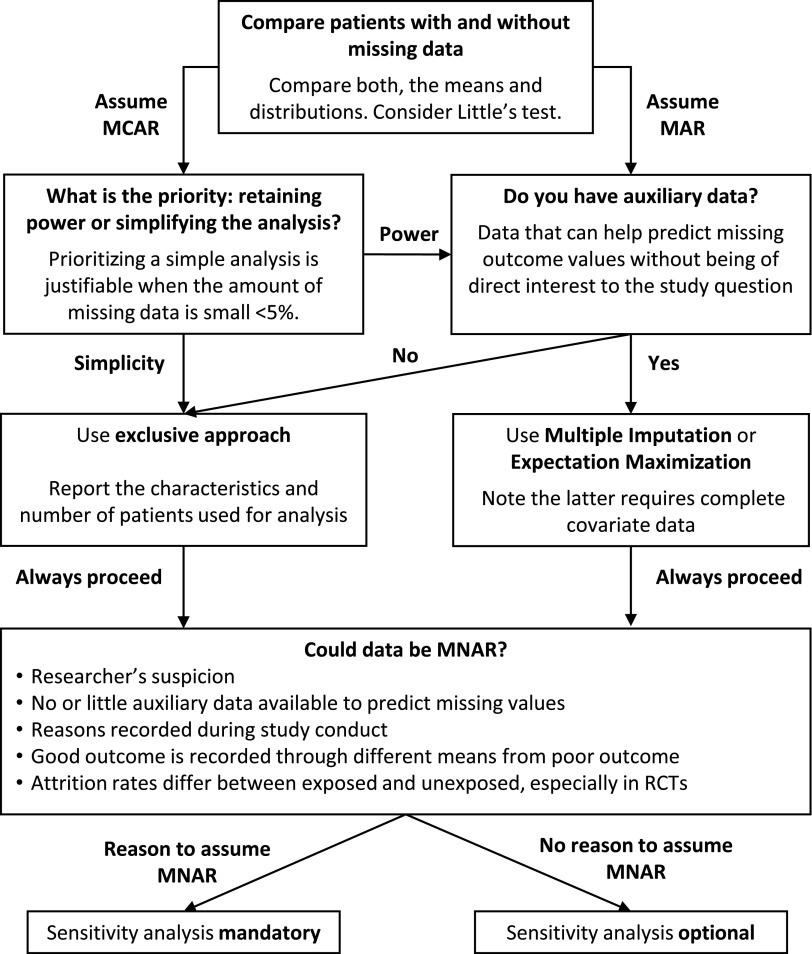
Decision aid for handling missing outcome data.

These shortcomings are understandable in the observational studies (the majority of TBI studies), because no guidance exists for the handling of missing data in this setting. However, guideline adherence was poor even among interventional studies, which highlights the need for improved awareness and more explicit guidance for the handling of missing outcome data within the TBI community, irrespective of study type. We therefore propose the following framework (Fig. 4).

When no difference exists between patients with and without available outcome data (MCAR assumed), exclusion or omission of lost patients from the analysis will not introduce bias. To decide if data are truly MCAR, it is insufficient to simply compare baseline means alone. For example, if both extremes of age are lost to follow-up, the mean age would remain unaffected. Therefore we recommend comparing distributions of the baseline characteristics, either by inspection of density plots or using a formal statistical test, such as Little's MCAR test.^24^ Little's test will distinguish between MCAR and MAR, but it cannot be used to exclude or identify MNAR. Whereas omission of patients who are truly MCAR will not introduce bias, it will nonetheless reduce power of studies. Therefore, we only recommend this strategy when the amount of missing data is very small, or alternative strategies are not possible because auxiliary data are unavailable (see below).

When missing patients are omitted (the number is small and MCAR is assumed), baseline data should be presented for patients successfully followed up, as described by the exclusive approach. This allows the reader to interpret the study results in the context of the patients who contributed to outcomes. In this review 76 (55%) of the 139 studies with missing outcome data presented baseline data for a larger cohort than the subgroup of patients with available outcomes (i.e., inclusive approach using “omission”). This should be avoided as it is not transparent and disrupts internal validity, especially when data are not MCAR.

An alternative approach, when data are MAR and sufficient information is available, is estimation of missing values using multiple imputation (MI) or advanced statistical techniques such as multi-level models (MLM).^25^ These techniques generate unbiased estimates for missing outcome values and therefore allow for inclusion of all patients and preservation of power. In addition, unlike single imputation where only a single value is estimated, they increase the standard errors to accurately reflect the uncertainty around estimation. Very few studies in our review used MI or MLM, which may reflect an unfamiliarity with these techniques. However, they have now been integrated in many of the mainstream statistics programs.

Both MI and MLM require some auxiliary information to estimate the missing values, that is, one or more variables that are correlated with the outcome of interest but are not directly relevant to the study question. For example, in a study investigating the effect of age at injury on GOS at 6 months, the use of age to estimate missing GOS values would be pointless. Ideally, the use of auxiliary outcome data, for example, GOS values measured at other time-points or a correlated outcome measure at the same time-point such as the Quality of Life after Brain Injury (QOLIBRI) Scale, could be used to estimate and impute the missing value. Additionally, covariate data such as GCS or study center can also be used to provide estimates that even more completely capture any correlations in the data.

When trying to choose between MI and MLM, one should be aware that MLM can utilize existing outcome data to predict outcomes when multiple time-points are used, provided covariate data are complete. Hence, MI may be needed to impute missing covariate data prior to multi-level modeling of outcome data itself. In the event of both missing covariate and outcome data, MI alone may be easier than multi-level modeling. Both MI and MLM have been shown to provide similar effect estimates and precision.^25^

In the event that data are suspected of being MNAR, that is, when follow-up depends on outcome itself and is not predictable using other observed information, it should be emphasised that MI and MLM cannot compensate for bias introduced. Unfortunately, there is no single test that can be used in isolation to reliably identify MNAR. We therefore recommend the following red flags that, in combination, can act as a suite of tools to suspect possible MNAR:

- The researchers' suspicion based on their knowledge of the field.
- The amount of auxiliary data available to predict missing values is low. Note that data that appear to be missing MNAR can become predictable (and thus MAR) if given enough auxiliary information. Therefore data from studies with multiple follow up time-points are unlikely to be MNAR, if the patient has attended at least one follow-up.
- Reasons recorded during the conduct of the study suggest MNAR, for example, patients reporting to withdraw from the studies for reasons related to their outcome status.
- If poor outcome is recorded from a different source than good outcome, and the source for poor outcome is more complete. For example, GOS 1 is sourced from the death registry, whereas GOS 2–5 are recorded in the outpatient clinic. This could cause MNAR unless circumvented by using “data source” as a variable in the MI or MLM.
- Attrition rates differ between exposed and unexposed, especially when patients were randomized to the exposure as in clinical trials.

When MNAR is suspected, a sensitivity analysis should be performed, as this can provide insight into the validity of any conclusions drawn. In the few studies that undertook a sensitivity analysis, imputation was compared with exclusion of missing values.^26–30^ A sensitivity analysis can specifically test any bias that the researcher is concerned about. For example, if one suspects that patients with good recovery are less inclined to return for follow-up, one could specifically impute more favorable outcomes for the lost patients to assess whether the conclusions still hold.

Our data support previous studies that flagged patient attrition as a common problem in TBI research.^1–3^ We have addressed the issue of handling missing outcome data with the aim of reducing bias and maintaining study power. Our framework is not designed to replace diligent follow-up. Ideally, studies should be designed in such a way as to minimize patient attrition in the first place. Previous work looking at patient-specific factors predicting dropout have identified that higher functional status at baseline and lower socioeconomic background reduce the likelihood of patients attending follow-up, whereas a higher severity of initial injury increases rates of follow-up. Further work should focus on features of study design that enable researchers to maximize follow-up.

As any analysis, our systematic review has limitations. First, due to the common language of all the reviewers being English we were restricted to studies published in this language. However, our data collection revealed that studies originated from a broad range of high- and low-income countries. Second, including only large cohorts (more than 100 TBI patients) may have selected for studies that were well funded and therefore had better follow-up rates than less well-funded studies. Therefore, the extent of missing data in TBI research may be even larger than estimated in this review. Finally, although we eliminated duplicate cohorts from our studies, we cannot guarantee exclusion of some patients participating in more than one cohort.

## Conclusion

Missing outcome data in TBI research occurs frequently, is reported inconsistently, and is handled suboptimally. We propose a framework to handle missing outcome data that is practical and accessible to the non-statistician.

## Supplementary Material

Supplemental data

Supplemental data
