# Supplementary material for: Handling of Missing Outcome Data in Traumatic Brain Injury Research: A Systematic Review
Source: J Neurotrauma. 2019 Sep 10;36(19):2743–52. doi: 10.1089/neu.2018.6216 (PMC6744946; doi:10.1089/neu.2018.6216)
Supplement: Supplemental data [file Supp_Appendix1.docx]

**Supplementary Appendix S1. Risk of Bias Assessment**

Risk of bias was assessed in accordance with the Cochrane Handbook for Systematic Reviews of Interventions^1^ using the Cochrane risk of bias tool for clinical trials^2^ and the Newcastle-Ottawa Scale^3^ for cohort. Briefly, the Cochrane risk of bias tool looks at seven areas in trial design and implementation with potential for the introduction of bias. The risk is assessed as high, low, or unclear. Similarly, the Newcastle-Ottawa Scale assesses the quality of non-randomized studies including cohorts, in eight domains using a star system. Each domain could score "1 star" depending on quality, with the exception of "comparability of cohorts" where 2 stars could be awarded when multiple confounders were controlled for.

Two reviewers assessed risk of bias independently for each study (two of SS, SR, TN). Disagreements were resolved through discussion and consensus with the third reviewer. Overall, the risk of bias in these studies was judged to be low–moderate.


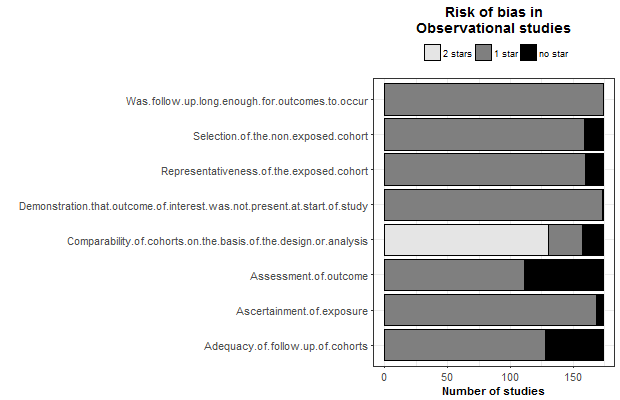


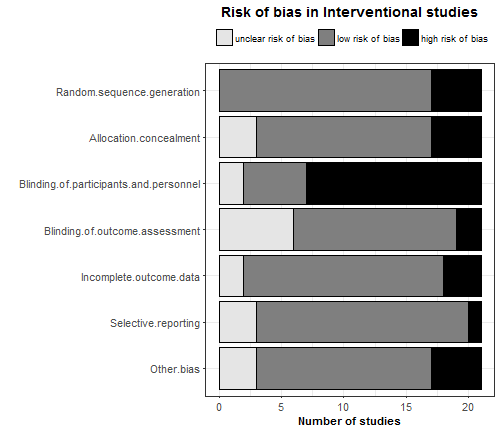


**<H1>References**

<REF>

1. Higgins, J., and Green, S. (2011). Cochrane Handbook for Systematic Reviews of Interventions - Version 5.1.0. Cochrane Collab., Appendix. www.cochrane-handbook.org (Last accessed September 1, 2017).

2. Higgins, J.P.T., Altman, D.G., Gøtzsche, P.C., Jüni, P., Moher, D., Oxman, A.D., Savović, J., Schulz, K.F., Weeks, L., and Sterne, J.A.C. (2011). The Cochrane Collaboration’s tool for assessing risk of bias in randomised trials. BMJ 343, d5928.

3. Wells, G., Shea, B., O’Connell, D., Peterson, J., Welch, V., Losos, M., and Tugwell, P. The Newcastle-Ottawa Scale (NOS) for assessing the quality of nonrandomised studies in meta-analyses. The Ottawa Hospital Research Institute. http://www.ohri.ca/programs/clinical_epidemiology/oxford.htm (Last accessed September 1, 2017).
