# Supplementary material for: Handling of Missing Outcome Data in Traumatic Brain Injury Research: A Systematic Review
Source: J Neurotrauma. 2019 Sep 10;36(19):2743–52. doi: 10.1089/neu.2018.6216 (PMC6744946; doi:10.1089/neu.2018.6216)
Supplement: Supplemental data [file Supp_Appendix2.docx]

**Supplementary Appendix S2. Overview of Included Studies**

| **ID** | **Author** | **Year of publication** | **Study design** | **Retro - vs Pro outcome collection** | **Number of follow up time points** | **Number of patients recruited** | **Number of patients lost to follow up** | **Number of patients remaining at the end of the study** | **Percentage of missing outcome data** | **Reporting pattern of missing outcome data** | **Approach to missing outcome data** | **Comparison of lost and retained patients** | **Comparison of attrition rates across groups** | **Technique for handling missing outcome data** |
| --- | --- | --- | --- | --- | --- | --- | --- | --- | --- | --- | --- | --- | --- | --- |
| 1 | Leong^1^ | 2013 | cohort | Pro | 1 | 172 | 72 | 100 | 42 | reported per follow up | exclusive | Descriptive statistics | not reported | exclusive approach |
| 3 | Khalili^2^ | 2017 | cohort | Pro | 2 | NA | NA | 152 | NA | reported but not quantified | inclusive | not compared | different | omission |
| 4 | MacDonald^3^ | 2016 | cohort | Pro | 1 | 337 | 138 | 199 | 41 | reported per follow up | inclusive | Descriptive statistics | similar | omission |
| 5 | Adams^4^ | 2017 | cohort | Pro | 1 | 601 | 0 | 601 | 0 | apparently complete data | no missing data | no missing data | no data missing | no missing data |
| 6 | Charry^5^ | 2016 | cohort | Retro | 1 | 106 | 0 | 106 | 0 | apparently complete data | no missing data | no missing data | no data missing | no missing data |
| 7 | Nielson^6^ | 2017 | cohort | Pro | 2 | 586 | 182 | 404 | 31 | reported per follow up | inclusive | not compared | not reported | omission |
| 11 | Agrawal^7^ | 2016 | cohort | Pro | 1 | 1345 | 444 | 901 | 33 | reported per follow up | inclusive | not compared | similar | omission |
| 12 | Khalili^8^ | 2017 | non-randomised trial | Pro | 2 | 336 | 29 | 307 | 9 | reported overall | exclusive | not compared | different | exclusive approach |
| 14 | Jourdan^9^ | 2013 | cohort | Pro | 1 | 257 | 123 | 134 | 48 | reported per follow up | exclusive | Statistical test | not reported | exclusive approach |
| 16 | Yousuf^10^ | 2015 | cohort | Pro | 1 | 150 | 0 | 150 | 0 | apparently complete data | no missing data | no missing data | no data missing | no missing data |
| 17 | Agrawal^11^ | 2015 | cohort | Retro | 1 | 166 | 65 | 101 | 39 | reported per follow up | inclusive | not compared | similar | omission |
| 19 | Al Nimer^12^ | 2015 | cohort | Retro | 1 | 182 | 0 | 182 | 0 | complete follow up declared | no missing data | no missing data | no data missing | no missing data |
| 22 | Andrews^13^ | 2015 | RCT (parallel) | Pro | 1 | 387 | 4 | 383 | 1 | reported per follow up | inclusive | not compared | similar | omission |
| 24 | Anglin^14^ | 2013 | cohort | Retro | 1 | 674 | 194 | 480 | 29 | reported per follow up | exclusive | not compared | not reported | exclusive approach |
| 25 | Arbour^15^ | 2016 | cohort | Pro | 1 | 181 | 38 | 143 | 21 | reported per follow up | inclusive | Descriptive statistics | different | omission |
| 26 | Badri^16^ | 2012 | cohort | Retro | 3 | 365 | 33 | 332 | 9 | reported per follow up | inclusive | not compared | similar | omission |
| 27 | Bao^17^ | 2014 | cohort | Retro | 1 | 355 | 0 | 355 | 0 | apparently complete data | no missing data | no missing data | no data missing | no missing data |
| 28 | Bertisch^18^ | 2016 | cohort | Retro | 3 | 399 | 271 | 128 | 68 | reported per follow up | inclusive | not compared | similar | omission |
| 30 | Bulger^19^ | 2012 | cohort | Pro | 1 | 1238 | 186 | 1052 | 15 | reported per follow up | inclusive | not compared | similar | multiple imputation |
| 32 | Cepeda^20^ | 2015 | cohort | Retro | 1 | 782 | 23 | 759 | 3 | reported per follow up | inclusive | not compared | not reported | omission |
| 34 | Charry^21^ | 2017 | cohort | Retro | 1 | 127 | 0 | 127 | 0 | apparently complete data | no missing data | no missing data | no data missing | no missing data |
| 36 | Chen^22^ | 2016 | cohort | Pro | 1 | 115 | 2 | 113 | 2 | reported per follow up | exclusive | not compared | not reported | exclusive approach |
| 37 | Chen^23^ | 2014 | cohort | Pro | 1 | 148 | 0 | 148 | 0 | apparently complete data | no missing data | no missing data | no data missing | no missing data |
| 38 | Cheng^24^ | 2017 | cohort | Pro | 2 | 251 | 65 | 186 | 26 | reported per follow up | inclusive | not compared | not reported | omission |
| 39 | Chestnut^25^ | 2012 | RCT (parallel) | Pro | 2 | 324 | 26 | 298 | 8 | reported per follow up | inclusive | Statistical test | similar | omission |
| 40 | Chiang^26^ | 2016 | cohort | Pro | 3 | 103 | 3 | 100 | 3 | reported overall | exclusive | not compared | not reported | exclusive approach |
| 41 | Cicuendez^27^ | 2017 | cohort | Pro | 1 | 264 | 0 | 264 | 0 | apparently complete data | no missing data | no missing data | no data missing | no missing data |
| 42 | Cipolle^28^ | 2014 | cohort | Pro | 1 | 164 | 25 | 139 | 15 | reported per follow up | inclusive | not compared | not reported | omission |
| 43 | Cnossen^29^ | 2017 | cohort | Pro | 1 | 503 | 91 | 412 | 18 | reported per follow up | inclusive | Statistical test | not reported | omission |
| 44 | Conley^30^ | 2014 | cohort | Pro | 3 | NA | NA | 336 | NA | reported but not quantified | inclusive | not compared | not reported | omission |
| 45 | Corral^31^ | 2012 | cohort | Retro | 1 | 224 | 0 | 224 | 0 | apparently complete data | no missing data | no missing data | no data missing | no missing data |
| 48 | Dahdah^32^ | 2014 | cohort | Pro | 1 | 6975 | 2302 | 4673 | 33 | reported per follow up | inclusive | not compared | not reported | omission |
| 49 | Dahdah^33^ | 2016 | cohort | Pro | 1 | 5505 | 1266 | 4239 | 23 | reported per follow up | inclusive | not compared | not reported | omission |
| 50 | Dams-O'Connor^34^ | 2013 | cohort | Pro | 2 | 586 | 0 | 586 | 0 | apparently complete data | no missing data | no missing data | no data missing | no missing data |
| 51 | Dams-O'Connor^35^ | 2015 | cohort | Pro | 3 | 4178 | 308 | 3870 | 7 | reported overall | exclusive | not compared | not reported | exclusive approach |
| 53 | Dardiotis^36^ | 2014 | cohort | Pro | 1 | 363 | 0 | 363 | 0 | apparently complete data | no missing data | no missing data | no data missing | no missing data |
| 54 | de Haan^37^ | 2017 | cohort | Retro | 1 | 127 | 0 | 127 | 0 | apparently complete data | no missing data | no missing data | no data missing | no missing data |
| 57 | de Koning^38^ | 2015 | cohort | Retro | 1 | 361 | 18 | 343 | 5 | reported per follow up | exclusive | Descriptive statistics | not reported | exclusive approach |
| 59 | Depreitere^39^ | 2014 | cohort | Pro | 1 | NA | NA | 180 | NA | reported but not quantified | exclusive | not compared | not reported | exclusive approach |
| 60 | Dhandapani^40^ | 2012 | cohort | Pro | 2 | 244 | 90 | 154 | 37 | reported per follow up | inclusive | not compared | not reported | omission |
| 61 | Dhandapani^41^ | 2015 | cohort | Pro | 1 | 344 | 24 | 320 | 7 | reported per follow up | inclusive | not compared | not reported | omission |
| 63 | DiBattista^42^ | 2016 | cohort | Pro | 1 | 166 | 0 | 166 | 0 | apparently complete data | no missing data | no missing data | no data missing | no missing data |
| 65 | Egea-Guerrero^43^ | 2017 | cohort | Pro | 1 | 340 | 50 | 290 | 15 | reported per follow up | exclusive | not compared | not reported | exclusive approach |
| 67 | Esnault^44^ | 2017 | cohort | Retro | 2 | 175 | 19 | 156 | 11 | reported per follow up | inclusive | not compared | different | omission |
| 68 | Fabbri^45^ | 2013 | cohort | Pro | 1 | 1558 | 343 | 1215 | 22 | reported per follow up | inclusive | not compared | not reported | omission |
| 69 | Failla^46^ | 2016 | cohort | Pro | 2 | 203 | 41 | 162 | 20 | reported per follow up | inclusive | not compared | not reported | omission |
| 71 | Firsching^47^ | 2015 | cohort | Retro | 1 | 157 | 0 | 157 | 0 | complete follow up declared | no missing data | no missing data | no data missing | no missing data |
| 72 | Forslund^48^ | 2017 | cohort | Pro | 3 | 133 | 12 | 121 | 9 | reported per follow up | inclusive | Statistical test | not reported | omission |
| 75 | Galanaud^49^ | 2012 | cohort | Pro | 1 | 115 | 10 | 105 | 9 | reported per follow up | exclusive | not compared | not reported | exclusive approach |
| 77 | Gao^50^ | 2014 | cohort | Pro | 1 | 128 | 0 | 128 | 0 | apparently complete data | no missing data | no missing data | no data missing | no missing data |
| 78 | Gao^51^ | 2013 | cohort | Retro | 1 | 127 | 79 | 48 | 62 | reported per follow up | inclusive | not compared | similar | omission |
| 79 | Garner^52^ | 2015 | RCT (parallel) | Pro | 1 | 375 | 34 | 341 | 9 | reported per follow up | inclusive | not compared | similar | single imputation |
| 80 | Garringer^53^ | 2013 | cohort | Pro | 1 | 110 | 0 | 110 | 0 | complete follow up declared | no missing data | no missing data | no data missing | no missing data |
| 82 | Godbolt^54^ | 2015 | cohort | Pro | 1 | 114 | 7 | 107 | 6 | reported per follow up | inclusive | not compared | not reported | omission |
| 85 | Goyal^55^ | 2013 | cohort | Pro | 1 | 160 | 24 | 136 | 15 | reported per follow up | inclusive | not compared | not reported | omission |
| 86 | Grauwmeijer^56^ | 2012 | cohort | Pro | 2 | 113 | 19 | 94 | 17 | reported per follow up | inclusive | Statistical test | not reported | omission |
| 87 | Gressot^57^ | 2014 | cohort | Pro | 1 | 119 | 4 | 115 | 3 | reported per follow up | inclusive | not compared | similar | omission |
| 88 | Griesdale^58^ | 2015 | cohort | Retro | 1 | 116 | 0 | 116 | 0 | apparently complete data | no missing data | no missing data | no data missing | no missing data |
| 90 | Guiza^59^ | 2017 | cohort | Pro | 1 | 259 | 8 | 251 | 3 | reported per follow up | inclusive | not compared | not reported | omission |
| 91 | Guiza^60^ | 2013 | cohort | Pro | 1 | NA | NA | 160 | NA | reported but not quantified | exclusive | Descriptive statistics | not reported | exclusive approach |
| 92 | Guo^61^ | 2017 | cohort | Retro | 1 | NA | NA | 147 | NA | reported but not quantified | exclusive | not compared | not reported | exclusive approach |
| 93 | Gupta^62^ | 2016 | cohort | Both | 4 | 400 | 84 | 316 | 21 | reported per follow up | inclusive | not compared | different | omission |
| 94 | Haagsma^63^ | 2015 | cohort | Retro | 2 | 1919 | 1631 | 288 | 85 | reported per follow up | inclusive | Statistical test | not reported | omission |
| 96 | Hamed^64^ | 2016 | cohort | Pro | 1 | 196 | 0 | 196 | 0 | apparently complete data | no missing data | no missing data | no data missing | no missing data |
| 97 | Han^65^ | 2014 | cohort | Pro | 1 | 300 | 0 | 300 | 0 | complete follow up declared | no missing data | no missing data | no data missing | no missing data |
| 98 | Harrison^66^ | 2013 | cohort | Pro | 1 | 3210 | 578 | 2632 | 18 | reported per follow up | inclusive | not compared | similar | multiple imputation |
| 101 | Hart^67^ | 2016 | cohort | Pro | 1 | 274 | 16 | 258 | 6 | reported per follow up | inclusive | Statistical test | similar | omission |
| 102 | Hatefi^68^ | 2016 | cohort | Pro | 1 | NA | NA | 725 | NA | reported but not quantified | exclusive | not compared | not reported | exclusive approach |
| 103 | Hellstrom^69^ | 2017 | cohort | Pro | 1 | 200 | 53 | 147 | 26 | reported per follow up | exclusive | not compared | not reported | exclusive approach |
| 104 | Henninger^70^ | 2014 | cohort | Retro | 2 | 148 | 50 | 98 | 34 | reported overall | inclusive | Statistical test | not reported | omission |
| 105 | Herrera-Melero^71^ | 2015 | cohort | Retro | 2 | 629 | 50 | 579 | 8 | reported per follow up | inclusive | not compared | not reported | omission |
| 109 | Honeybul^72^ | 2016 | cohort | Both | 1 | 319 | 0 | 319 | 0 | apparently complete data | no missing data | no missing data | no data missing | no missing data |
| 112 | Hua^73^ | 2014 | RCT (parallel) | Pro | 1 | 122 | 4 | 118 | 3 | reported per follow up | inclusive | not compared | similar | omission |
| 113 | Huang^74^ | 2013 | cohort | Retro | 1 | 118 | 13 | 105 | 11 | reported per follow up | exclusive | not compared | not reported | exclusive approach |
| 114 | Hudak^75^ | 2012 | cohort | Pro | 1 | 805 | 339 | 466 | 42 | reported per follow up | exclusive | not compared | not reported | exclusive approach |
| 115 | Hutchinson^76^ | 2016 | RCT (parallel) | Pro | 2 | 403 | 28 | 375 | 7 | reported per follow up | inclusive | not compared | similar | omission |
| 116 | Iaccarino^77^ | 2014 | cohort | Pro | 1 | NA | NA | 352 | NA | reported but not quantified | exclusive | not compared | not reported | exclusive approach |
| 117 | Jacobs^78^ | 2013 | cohort | Pro | 1 | 700 | 133 | 567 | 19 | reported per follow up | inclusive | not compared | similar | omission plus LOCF |
| 118 | Jin^79^ | 2012 | cohort | Pro | 1 | 117 | 3 | 114 | 3 | reported per follow up | exclusive | not compared | not reported | exclusive approach |
| 119 | Junaid^80^ | 2016 | cohort | Retro | 1 | 1056 | 0 | 1056 | 0 | apparently complete data | no missing data | no missing data | no data missing | no missing data |
| 120 | Kaloostian^81^ | 2012 | cohort | Pro | 4 | 120 | 35 | 85 | 29 | reported per follow up | inclusive | not compared | not reported | omission |
| 123 | Kasprowicz^82^ | 2016 | cohort | Pro | 2 | 162 | 3 | 159 | 2 | reported per follow up | inclusive | not compared | not reported | omission |
| 124 | Katsnelson^83^ | 2012 | cohort | Retro | 1 | 101 | 0 | 101 | 0 | apparently complete data | no missing data | no missing data | no data missing | no missing data |
| 125 | Kesinger^84^ | 2015 | cohort | Retro | 3 | NA | NA | 141 | NA | reported but not quantified | inclusive | Statistical test | similar | omission |
| 126 | Khalili^85^ | 2017 | cohort | Retro | 3 | 142 | 0 | 142 | 0 | apparently complete data | no missing data | no missing data | no data missing | no missing data |
| 127 | Khalili^86^ | 2017 | cohort | Retro | 3 | NA | NA | 129 | NA | reported but not quantified | exclusive | not compared | not reported | exclusive approach |
| 128 | Khalili^87^ | 2016 | cohort | Pro | 1 | NA | NA | 248 | NA | reported but not quantified | exclusive | not compared | not reported | exclusive approach |
| 129 | Kim^88^ | 2012 | cohort | Retro | 1 | 136 | 0 | 136 | 0 | apparently complete data | no missing data | no missing data | no data missing | no missing data |
| 131 | Korley^89^ | 2016 | cohort | Pro | 1 | 159 | 49 | 110 | 31 | reported per follow up | inclusive | Statistical test | not reported | omission |
| 132 | Kumar^90^ | 2015 | cohort | Pro | 2 | 114 | 22 | 92 | 19 | reported per follow up | inclusive | not compared | not reported | omission |
| 137 | Leal-Noval^91^ | 2016 | cohort | Pro | 2 | 309 | 46 | 263 | 15 | reported per follow up | inclusive | Statistical test | not reported | omission |
| 138 | Lecky^92^ | 2017 | RCT (cluster) | Pro | 1 | 293 | 234 | 59 | 80 | reported per follow up | inclusive | not compared | similar | analysis abandoned |
| 141 | Leitgeb^93^ | 2013 | cohort | Retro | 1 | 767 | 31 | 736 | 4 | reported per follow up | inclusive | not compared | similar | omission |
| 142 | Leitgeb^94^ | 2012 | cohort | Retro | 1 | 311 | 40 | 271 | 13 | reported per follow up | inclusive | not compared | similar | single imputation |
| 144 | Lewis^95^ | 2012 | cohort | Pro | 1 | 187 | 0 | 187 | 0 | apparently complete data | no missing data | no missing data | no data missing | no missing data |
| 145 | Li^96^ | 2012 | RCT (parallel) | Pro | 1 | 182 | 0 | 182 | 0 | apparently complete data | no missing data | no missing data | no data missing | no missing data |
| 146 | Li^97^ | 2016 | cohort | Pro | 1 | 237 | 0 | 237 | 0 | apparently complete data | no missing data | no missing data | no data missing | no missing data |
| 147 | Li^98^ | 2016 | RCT (parallel) | Pro | 1 | 159 | 3 | 156 | 2 | reported per follow up | inclusive | not compared | different | omission |
| 148 | Limpastan^99^ | 2013 | cohort | Retro | 2 | 159 | 10 | 149 | 6 | reported per follow up | inclusive | not compared | not reported | omission |
| 154 | Lu^100^ | 2015 | cohort | Retro | 1 | 118 | 3 | 115 | 3 | reported per follow up | exclusive | not compared | not reported | exclusive approach |
| 155 | Lupi^101^ | 2014 | cohort | Pro | 3 | 105 | 0 | 105 | 0 | apparently complete data | no missing data | no missing data | no data missing | no missing data |
| 157 | Maekawa^102^ | 2015 | RCT (parallel) | Pro | 1 | 148 | 6 | 142 | 4 | reported per follow up | inclusive | not compared | similar | omission |
| 158 | Majdan^103^ | 2015 | cohort | Retro | 1 | 683 | 82 | 601 | 12 | reported per follow up | inclusive | not compared | different | omission |
| 159 | Majdan^104^ | 2014 | cohort | Retro | 2 | 778 | 233 | 545 | 30 | reported per follow up | inclusive | not compared | not reported | omission |
| 160 | Matsukawa^105^ | 2013 | cohort | Retro | 1 | 419 | 0 | 419 | 0 | apparently complete data | no missing data | no missing data | no data missing | no missing data |
| 161 | Matsukawa^106^ | 2012 | cohort | Retro | 1 | 371 | 19 | 352 | 5 | reported per follow up | inclusive | not compared | not reported | omission |
| 162 | Matsushima^107^ | 2012 | cohort | Pro | 1 | 109 | 0 | 109 | 0 | apparently complete data | no missing data | no missing data | no data missing | no missing data |
| 163 | Mauritz^108^ | 2014 | cohort | Pro | 1 | 852 | 0 | 852 | 0 | apparently complete data | no missing data | no missing data | no data missing | no missing data |
| 167 | McNett^109^ | 2016 | cohort | Pro | 2 | 138 | 45 | 93 | 33 | reported overall | inclusive | not compared | not reported | omission |
| 168 | Mendelow^110^ | 2015 | RCT (parallel) | Pro | 1 | 170 | 2 | 168 | 1 | reported per follow up | inclusive | not compared | similar | omission |
| 169 | Merzo^111^ | 2016 | cohort | Retro | 1 | 284 | 17 | 267 | 6 | reported per follow up | inclusive | not compared | different | omission |
| 170 | Moen^112^ | 2014 | cohort | Pro | 1 | 128 | 4 | 124 | 3 | reported per follow up | inclusive | not compared | similar | omission plus LOCF |
| 172 | Muniven-katappa^113^ | 2013 | cohort | Pro | 1 | 108 | 33 | 75 | 31 | reported per follow up | inclusive | not compared | similar | omission |
| 173 | Nelson^114^ | 2012 | cohort | Pro | 3 | 789 | 289 | 500 | 37 | reported overall | inclusive | not compared | not reported | single imputation |
| 175 | NICE SugarStudy^115^ | 2015 | RCT (parallel) | Pro | 1 | 391 | 74 | 317 | 19 | reported per follow up | inclusive | not compared | similar | omission |
| 176 | Nichol^116^ | 2015 | RCT (parallel) | Pro | 1 | 606 | 12 | 594 | 2 | reported per follow up | inclusive | not compared | similar | omission |
| 177 | Nishijima^117^ | 2015 | cohort | Retro | 1 | 225 | 37 | 188 | 16 | reported per follow up | exclusive | Statistical test | not reported | exclusive approach |
| 179 | Osier^118^ | 2017 | cohort | Pro | 3 | 397 | 70 | 327 | 18 | reported overall | inclusive | not compared | not reported | omission |
| 180 | Ostberg^119^ | 2014 | cohort | Pro | 1 | 1022 | 361 | 661 | 35 | reported per follow up | exclusive | Statistical test | not reported | exclusive approach |
| 181 | Ozyurt^120^ | 2015 | cohort | Retro | 1 | 104 | 3 | 101 | 3 | reported per follow up | exclusive | not compared | not reported | exclusive approach |
| 182 | Pakkanen^121^ | 2016 | cohort | Retro | 1 | 458 | 5 | 453 | 1 | reported per follow up | inclusive | not compared | different | omission plus LOCF |
| 183 | Pan^122^ | 2015 | cohort | Pro | 1 | 130 | 2 | 128 | 2 | reported per follow up | exclusive | not compared | not reported | exclusive approach |
| 184 | Panczykowski^123^ | 2012 | cohort | Pro | 1 | 646 | 59 | 587 | 9 | reported per follow up | exclusive | not compared | similar | exclusive approach |
| 185 | Patel^124^ | 2015 | cohort | Retro | 1 | 640 | 410 | 230 | 64 | reported per follow up | exclusive | Description in text | different | exclusive approach |
| 186 | Ponce^125^ | 2012 | cohort | Retro | 1 | NA | NA | 405 | NA | reported but not quantified | inclusive | not compared | not reported | single imputation |
| 187 | Prathep^126^ | 2017 | cohort | Retro | 4 | 200 | 170 | 30 | 85 | reported per follow up | inclusive | Description in text | not reported | omission |
| 188 | Prieto-Palomino^127^ | 2016 | cohort | Pro | 1 | 531 | 37 | 494 | 7 | reported per follow up | inclusive | not compared | not reported | omission |
| 189 | Qiu^128^ | 2012 | cohort | Pro | 1 | 142 | 0 | 142 | 0 | apparently complete data | no missing data | no missing data | no data missing | no missing data |
| 190 | Rahmani^129^ | 2016 | cohort | Pro | 2 | 150 | 0 | 150 | 0 | apparently complete data | no missing data | no missing data | no data missing | no missing data |
| 191 | Raj^130^ | 2014 | cohort | Retro | 1 | 890 | 44 | 846 | 5 | reported per follow up | inclusive | not compared | similar | omission |
| 195 | Rizoli^131^ | 2017 | cohort | Pro | 1 | 183 | 9 | 174 | 5 | reported per follow up | exclusive | not compared | not reported | exclusive approach |
| 197 | Robertson^132^ | 2014 | RCT (parallel) | Pro | 1 | 200 | 18 | 182 | 9 | reported per follow up | inclusive | not compared | similar | multiple imputation |
| 198 | Roozenbeek^133^ | 2012 | cohort | Pro | 1 | 2200 | 38 | 2162 | 2 | reported per follow up | exclusive | not compared | not reported | exclusive approach |
| 200 | Rundhaug^134^ | 2015 | cohort | Pro | 1 | 222 | 12 | 210 | 5 | reported per follow up | inclusive | not compared | not reported | omission plus LOCF |
| 201 | Santarsieri^135^ | 2014 | cohort | Pro | 2 | NA | NA | 130 | NA | reported but not quantified | inclusive | not compared | not reported | omission |
| 202 | Scheenen^136^ | 2016 | cohort | Pro | 1 | 528 | 227 | 301 | 43 | reported per follow up | inclusive | Statistical test | similar | omission |
| 204 | Scholten^137^ | 2015 | cohort | Pro | 2 | 2286 | 1897 | 389 | 83 | reported per follow up | inclusive | Statistical test | similar | omission |
| 205 | Sekhon^138^ | 2017 | Before-After Study | Pro | 1 | NA | NA | 113 | NA | reported but not quantified | exclusive | not compared | not reported | exclusive approach |
| 206 | Sherif^139^ | 2012 | cohort | Retro | 1 | 158 | 21 | 137 | 13 | reported per follow up | inclusive | not compared | not reported | omission |
| 207 | Shi^140^ | 2015 | cohort | Retro | 1 | 172 | 0 | 172 | 0 | apparently complete data | no missing data | no missing data | no data missing | no missing data |
| 208 | Shimoda^141^ | 2014 | cohort | Retro | 1 | 1123 | 235 | 888 | 21 | reported per follow up | exclusive | not compared | not reported | exclusive approach |
| 209 | Siddiqui^142^ | 2015 | cohort | Pro | 1 | 100 | 0 | 100 | 0 | apparently complete data | no missing data | no missing data | no data missing | no missing data |
| 210 | Sigurdardottir^143^ | 2016 | cohort | Pro | 1 | 172 | 43 | 129 | 25 | reported per follow up | exclusive | not compared | not reported | exclusive approach |
| 213 | Sinha^144^ | 2015 | cohort | Pro | 2 | 1293 | 403 | 890 | 31 | reported overall | inclusive | not compared | not reported | omission plus LOCF |
| 214 | Skolnick^145^ | 2014 | RCT (parallel) | Pro | 2 | 1179 | 47 | 1132 | 4 | reported per follow up | inclusive | not compared | similar | single imputation |
| 216 | Soberg^146^ | 2017 | cohort | Pro | 1 | 381 | 38 | 343 | 10 | reported per follow up | inclusive | not compared | not reported | omission |
| 218 | Stein^147^ | 2013 | cohort | Pro | 1 | 191 | 25 | 166 | 13 | reported per follow up | inclusive | Statistical test | not reported | omission |
| 220 | Stocchetti^148^ | 2012 | cohort | Pro | 1 | 1366 | 96 | 1270 | 7 | reported per follow up | inclusive | Description in text | not reported | omission |
| 222 | Sun^149^ | 2016 | cohort | Retro | 1 | 136 | 0 | 136 | 0 | apparently complete data | no missing data | no missing data | no data missing | no missing data |
| 223 | Sveen^150^ | 2015 | cohort | Pro | 1 | 148 | 0 | 148 | 0 | complete follow up declared | no missing data | no missing data | no data missing | no missing data |
| 226 | Takala^151^ | 2016 | cohort | Pro | 1 | 324 | 26 | 298 | 8 | reported per follow up | inclusive | not compared | not reported | omission plus LOCF |
| 227 | Taw^152^ | 2012 | cohort | Retro | 2 | 116 | 0 | 116 | 0 | apparently complete data | no missing data | no missing data | no data missing | no missing data |
| 228 | Theadom^153^ | 2015 | cohort | Pro | 1 | 378 | 250 | 128 | 66 | reported per follow up | inclusive | Statistical test | similar | omission |
| 230 | Thelin^154^ | 2014 | cohort | Retro | 1 | 250 | 0 | 250 | 0 | apparently complete data | no missing data | no missing data | no data missing | no missing data |
| 232 | Thelin^155^ | 2017 | cohort | Retro | 1 | 1204 | 89 | 1115 | 7 | reported per follow up | exclusive | not compared | not reported | exclusive approach |
| 233 | van der Horn^156^ | 2013 | cohort | Pro | 2 | 242 | 0 | 242 | 0 | apparently complete data | no missing data | no missing data | no data missing | no missing data |
| 234 | van der Naalt^157^ | 2017 | cohort | Pro | 1 | 1151 | 478 | 673 | 42 | reported per follow up | inclusive | Descriptive statistics | not reported | omission |
| 235 | Vathanalaoha^158^ | 2017 | cohort | Retro | 1 | 103 | 0 | 103 | 0 | apparently complete data | no missing data | no missing data | no data missing | no missing data |
| 237 | Wagner^159^ | 2012 | cohort | Pro | 2 | 129 | 8 | 121 | 6 | reported per follow up | inclusive | not compared | different | omission |
| 238 | Walker^160^ | 2015 | cohort | Pro | 1 | 9229 | 2046 | 7183 | 22 | reported per follow up | exclusive | not compared | not reported | exclusive approach |
| 240 | Wan^161^ | 2017 | cohort | Retro | 1 | 137 | 0 | 137 | 0 | apparently complete data | no missing data | no missing data | no data missing | no missing data |
| 243 | Wang^162^ | 2014 | cohort | Retro | 1 | 176 | 0 | 176 | 0 | apparently complete data | no missing data | no missing data | no data missing | no missing data |
| 244 | Wang^163^ | 2015 | cohort | Pro | 1 | 182 | 0 | 182 | 0 | complete follow up declared | no missing data | no missing data | no data missing | no missing data |
| 245 | Waqas^164^ | 2016 | cohort | Retro | 1 | NA | NA | 117 | NA | reported but not quantified | exclusive | not compared | not reported | exclusive approach |
| 246 | Waqas^165^ | 2016 | cohort | Retro | 1 | 197 | 8 | 189 | 4 | reported per follow up | inclusive | not compared | not reported | omission plus LOCF |
| 247 | Waters^166^ | 2013 | cohort | Retro | 1 | 942 | 5 | 937 | 1 | reported per follow up | exclusive | not compared | not reported | exclusive approach |
| 249 | Weisbrod^167^ | 2012 | cohort | Retro | 4 | 137 | 59 | 78 | 43 | reported per follow up | inclusive | not compared | not reported | omission |
| 250 | Willmott^168^ | 2014 | cohort | Pro | 5 | 412 | 362 | 50 | 88 | reported overall | inclusive | Statistical test | not reported | omission |
| 251 | Willmott^169^ | 2014 | cohort | Pro | 2 | 223 | 91 | 132 | 41 | reported per follow up | inclusive | not compared | similar | omission |
| 252 | Wilson^170^ | 2017 | cohort | Pro | 1 | 761 | 0 | 761 | 0 | complete follow up declared | no missing data | no missing data | no data missing | no missing data |
| 253 | Woischneck^171^ | 2017 | cohort | Pro | 1 | 140 | 0 | 140 | 0 | apparently complete data | no missing data | no missing data | no data missing | no missing data |
| 254 | Woischneck^172^ | 2015 | cohort | Pro | 1 | 120 | 0 | 120 | 0 | apparently complete data | no missing data | no missing data | no data missing | no missing data |
| 256 | Wright^173^ | 2014 | RCT (parallel) | Pro | 1 | 882 | 53 | 829 | 6 | reported per follow up | inclusive | not compared | similar | multiple imputation |
| 258 | Xu^174^ | 2014 | RCT (parallel) | Pro | 1 | NA | NA | 169 | NA | reported but not quantified | exclusive | not compared | not reported | exclusive approach |
| 259 | Xu^175^ | 2016 | non-randomised trial | Pro | 1 | 108 | 1 | 107 | 1 | reported per follow up | inclusive | not compared | similar | omission |
| 261 | Yang^176^ | 2014 | cohort | Retro | 1 | 118 | 0 | 118 | 0 | apparently complete data | no missing data | no missing data | no data missing | no missing data |
| 262 | Yang^177^ | 2017 | cohort | Pro | 1 | 108 | 0 | 108 | 0 | apparently complete data | no missing data | no missing data | no data missing | no missing data |
| 263 | Yao^178^ | 2017 | cohort | Retro | 1 | 352 | 50 | 302 | 14 | reported per follow up | exclusive | not compared | not reported | exclusive approach |
| 265 | You^179^ | 2016 | cohort | Pro | 1 | 176 | 10 | 166 | 6 | reported per follow up | exclusive | not compared | similar | exclusive approach |
| 266 | Yu^180^ | 2012 | cohort | Pro | 1 | 698 | 0 | 698 | 0 | complete follow up declared | no missing data | no missing data | no data missing | no missing data |
| 267 | Yu^181^ | 2012 | cohort | Pro | 1 | 110 | 4 | 106 | 4 | reported per follow up | exclusive | not compared | not reported | exclusive approach |
| 268 | Gaetani^182^ | 2012 | cohort | Retro | 1 | 103 | 0 | 103 | 0 | complete follow up declared | no missing data | no missing data | no data missing | no missing data |
| 271 | Yu^183^ | 2015 | cohort | Retro | 1 | 223 | 11 | 212 | 5 | reported per follow up | inclusive | not compared | different | omission |
| 272 | Yu^184^ | 2015 | cohort | Pro | 1 | 122 | 0 | 122 | 0 | apparently complete data | no missing data | no missing data | no data missing | no missing data |
| 273 | Yuan^185^ | 2016 | cohort | Pro | 1 | 499 | 17 | 482 | 3 | reported per follow up | exclusive | not compared | not reported | exclusive approach |
| 274 | Yuan^186^ | 2015 | cohort | Retro | 1 | 1480 | 37 | 1443 | 2 | reported per follow up | exclusive | not compared | not reported | exclusive approach |
| 279 | Zador^187^ | 2016 | cohort | Pro | 1 | 7398 | 453 | 6945 | 6 | reported per follow up | exclusive | Descriptive statistics | not reported | exclusive approach |
| 280 | Zafonte^188^ | 2012 | RCT (parallel) | Pro | 2 | 1213 | 315 | 898 | 26 | reported per follow up | inclusive | not compared | similar | omission |
| 283 | Zeng^189^ | 2013 | non-randomised trial | Pro | 1 | 168 | 0 | 168 | 0 | apparently complete data | no missing data | no missing data | no data missing | no missing data |
| 284 | Zhang^190^ | 2017 | cohort | Retro | 1 | 623 | 5 | 618 | 1 | reported per follow up | exclusive | not compared | not reported | exclusive approach |
| 285 | Zhang^191^ | 2014 | cohort | Pro | 1 | 102 | 0 | 102 | 0 | apparently complete data | no missing data | no missing data | no data missing | no missing data |
| 286 | Zhao^192^ | 2017 | RCT (parallel) | Pro | 2 | 128 | 0 | 128 | 0 | apparently complete data | no missing data | no missing data | no data missing | no missing data |
| 287 | Zhao^193^ | 2017 | cohort | Pro | 1 | 222 | 3 | 219 | 1 | reported per follow up | exclusive | not compared | not reported | exclusive approach |
| 288 | Zheng^194^ | 2015 | cohort | Pro | 1 | 164 | 0 | 164 | 0 | complete follow up declared | no missing data | no missing data | no data missing | no missing data |
| 292 | Walder^195^ | 2013 | cohort | Pro | 2 | 921 | 249 | 672 | 27 | reported per follow up | inclusive | not compared | not reported | omission |

1. Leong, B.K., Mazlan, M., Abd Rahim, R.B., and Ganesan, D. (2013). Concomitant injuries and its influence on functional outcome after traumatic brain injury. Disabil. Rehabil. 35, 1546–1551.

2. Khalili, H., Paydar, S., Safari, R., Arasteh, P., Niakan, A., and Abolhasani Foroughi, A. (2017). Experience with Traumatic Brain Injury: Is Early Tracheostomy Associated with Better Prognosis?. World Neurosurg. 103, 88–93.

3. MacDonald, C., Johnson, A., Wierzechowski, L., Kassner, E., Stewart, T., Nelson, E., Werner, N., Adam, O., Rivet, D., Flaherty, S., Oh, J., Zonies, D., Fang, R., and Brody, D. (2017). Outcome Trends after US Military Concussive Traumatic Brain Injury. J. Neurotrauma 34, 2206–2219.

4. Adams, H., Donnelly, J., Czosnyka, M., Kolias, A.G., Helmy, A., Menon, D.K., Smielewski, P., and Hutchinson, P.J. (2017). Temporal profile of intracranial pressure and cerebrovascular reactivity in severe traumatic brain injury and association with fatal outcome: An observational study. PLoS Med. 14, e1002353.

5. Charry, J.D., Rubiano, A.M., Nikas, C.V., Ortiz, V., Puyana, J.C., Carney, N., and Adelson, P.D. (2016). Results of early cranial decompression as an initial approach for damage control therapy in severe traumatic brain injury in a hospital with limited resources. J. Neurosci. Rural Pract. 7, 7–12.

6. Nielson, J.L., Cooper, S.R., Yue, J.K., Sorani, M.D., Inoue, T., Yuh, E.L., Mukherjee, P., Petrossian, T.C., Paquette, J., Lum, P.Y., Carlsson, G.E., Vassar, M.J., Lingsma, H.F., Gordon, W.A., Valadka, A.B., Okonkwo, D.O., Manley, G.T., Ferguson, A.R., Adeoye, O.M., Badjatia, N., Boase, K.D., Bodien-Guller, Y., Bullock, M.R., Chesnut, R.M., Corrigan, J.D., Crawford, K.L., Diaz-Arrastia, R., Dikmen, S.S., Duhaime, A.-C., Ellenbogen, R.G., Ezekiel, F., Feeser, V.R., Giacino, J.T., Goldman, D.P., Gonzales, L., Gopinath, S.P., Gullapalli, R.P., Hemphill, J.C., Hotz, G.A., Kramer, J.H., Levin, H., Lindsell, C.J., Machamer, J., Madden, C., Markowitz, A.J., Martin, A., Mathern, B.E., McAllister, T.W., McCrea, M.A., Merchant, R.E., Noel, F., Perl, D.P., Puccio, A.M., Rabinowitz, A.M., Robertson, C.S., Rosand, V., Sander, A.M., Satris, G., Schnyer, D.M., Seabury, S.A., Sergot, P., Sherer, M., Stein, D.M., Stein, M.B., Taylor, S.R., Temkin, N.R., Toga, A.W., Christine Turtzo, L., Vespa, P.M., Wang, K.K., Zafonte, R., and Zhang, Z. (2017). Uncovering precision phenotype-biomarker associations in traumatic brain injury using topological data analysis. PLoS One 12, e0169490.

7. Agrawal, D., Raghavendran, K., Schaubel, D.E., Mishra, M.C., and Rajajee, V. (2016). A propensity score analysis of the impact of invasive intracranial pressure monitoring on outcomes after severe traumatic brain injury. J. Neurotrauma 33, 853–858.

8. Khalili, H., Ahl, R., Cao, Y., Paydar, S., Sjolin, G., Niakan, A., Dabiri, G., and Mohseni, S. (2017). Early selenium treatment for traumatic brain injury: Does it improve survival and functional outcome? Injury 48, 1922–1926.

9. Jourdan, C., Bosserelle, V., Azerad, S., Ghout, I., Bayen, E., Aegerter, P., Weiss, J.J., Mateo, J., Lescot, T., Vigue, B., Tazarourte, K., Pradat-Diehl, P., Azouvi, P., study, members of the steering committee of the P.-T., and Frederic Ricard-Hibon, El Sayed, Cabaret, Le Quellec, Devaux, Sebbah, Cuvier, Lambros, Binda, Rakotonirina, Hazan, Briole, Parpet, Faggianelli, Touitou, Nguyen, Letarnec, Soupizet, Chollet-Xemard, Adnet, Luis, Lapostolle, Hennequin, Beruben, Telion, Kim, M. (2013). Predictive factors for 1-year outcome of a cohort of patients with severe traumatic brain injury (TBI): results from the PariS-TBI study. Brain Inj. 27, 1000–1007.

10. Yousuf, A., Khursheed, N., Rasool, I., Kundal, V., Jeelani, H., and Afroze, D. (2015). Genetic Variation of ApoE Gene in Ethnic Kashmiri Population and Its Association with Outcome After Traumatic Brain Injury. J. Mol. Neurosci. 56, 597–601.

11. Agrawal, D., Singh, P.K., Sinha, S., Gupta, D.K., Satyarthee, G.D., and Misra, M.C. (2015). Remaining unconscious: The burden of traumatic brain injuries in India. J. Neurosci. Rural Pract. 6, 520–522.

12. Al Nimer, F., Thelin, E., Nystrom, H., Dring, A.M., Svenningsson, A., Piehl, F., Nelson, D.W., and Bellander, B.-M. (2015). Comparative Assessment of the Prognostic Value of Biomarkers in Traumatic Brain Injury Reveals an Independent Role for Serum Levels of Neurofilament Light. PLoS One 10, e0132177.

13. Andrews, P.J.D., Sinclair, H.L., Rodriguez, A., Harris, B.A., Battison, C.G., Rhodes, J.K.J., Murray, G.D., Collaborators, E.T., and Noble D Taylor J, Addison J, Wallis C, Paterson R, Harris G, Mullan B, Quinn V, Bannon L, Gordon A, Templeton M, Wilson R, Thomas E, McMillan H, Tantam K, Gratrix A, Smith N, Martinson V, Barrera-Groba C, Littlejohn I, Spurling G, Hopkins P, Casboult S, G.S. (2015). Hypothermia for Intracranial Hypertension after Traumatic Brain Injury. N. Engl. J. Med. 373, 2403–2412.

14. Anglin, C.O., Spence, J.S., Warner, M.A., Paliotta, C., Harper, C., Moore, C., Sarode, R., Madden, C., and Diaz-Arrastia, R. (2013). Effects of platelet and plasma transfusion on outcome in traumatic brain injury patients with moderate bleeding diatheses. J. Neurosurg. 118, 676–686.

15. Arbour, C., Baril, A.-A., Westwick, H.J., Potvin, M.-J., Gilbert, D., Giguere, J.-F., Lavigne, G.J., Desautels, A., Bernard, F., Laureys, S., and Gosselin, N. (2016). Visual Fixation in the ICU: A Strong Predictor of Long-Term Recovery After Moderate-to-Severe Traumatic Brain Injury. Crit. Care Med. 44, e1186–e1193.

16. Badri, S., Chen, J., Barber, J., Temkin, N.R., Dikmen, S.S., Chesnut, R.M., Deem, S., Yanez, N.D., and Treggiari, M.M. (2012). Mortality and long-term functional outcome associated with intracranial pressure after traumatic brain injury. Intensive Care Med. 38, 1800–1809.

17. Bao, L., Chen, D., Ding, L., Ling, W., and Xu, F. (2014). Fever burden is an independent predictor for prognosis of traumatic brain injury. PLoS One 9, e90956.

18. Bertisch, H., Krellman, J.W., Bergquist, T.F., Dreer, L.E., Ellois, V., and Bushnik, T. (2016). Characteristics of Firearm Brain Injury Survivors in the Traumatic Brain Injury Model Systems (TBIMS) National Database: A Comparison of Assault and Self-Inflicted Injury Survivors. Arch. Phys. Med. Rehabil. .

19. Bulger, E.M., Guffey, D., Guyette, F.X., MacDonald, R.D., Brasel, K., Kerby, J.D., Minei, J.P., Warden, C., Rizoli, S., Morrison, L.J., Nichol, G., and Investigators, R.O.C. (2012). Impact of prehospital mode of transport after severe injury: a multicenter evaluation from the Resuscitation Outcomes Consortium. J. Trauma Acute Care Surg. 72, 567–803.

20. Cepeda, S., Gomez, P.A., Castano-Leon, A.M., Martinez-Perez, R., Munarriz, P.M., and Lagares, A. (2015). Traumatic Intracerebral Hemorrhage: Risk Factors Associated with Progression. J. Neurotrauma 32, 1246–1253.

21. Charry, J.D., Tejada, J.H., Pinzon, M.A., Tejada, W.A., Ochoa, J.D., Falla, M., Tovar, J.H., Cuellar-Bahamon, A.M., and Solano, J.P. (2017). Predicted Unfavorable Neurologic Outcome Is Overestimated by the Marshall Computed Tomography Score, Corticosteroid Randomization After Significant Head Injury (CRASH), and International Mission for Prognosis and Analysis of Clinical Trials in Traumatic B. World Neurosurg. 101, 554–558.

22. Chen, Q.-H., Lin, D., Zhou, J., and Deng, G. (2016). Role of signal peptide-Cub-Egf domain-containing protein-1 in serum as a predictive biomarker of outcome after severe traumatic brain injury. Clin. Chim. Acta. 456, 63–66.

23. Chen, T.-J., Fu, Q.-Y., and Wu, W.-Q. (2014). Plasma levels of adrenomedullin in patients with traumatic brain injury: potential contribution to prognosis. Peptides 56, 146–150.

24. Cheng, K., Bassil, R., Carandang, R., Hall, W., and Muehlschlegel, S. (2017). The Estimated Verbal GCS Subscore in Intubated Traumatic Brain Injury Patients: Is it Really Better?. J. Neurotrauma 34, 1603–1609.

25. Chesnut, R.M., Temkin, N., Carney, N., Dikmen, S., Rondina, C., Videtta, W., Petroni, G., Lujan, S., Pridgeon, J., Barber, J., Machamer, J., Chaddock, K., Celix, J.M., Cherner, M., Hendrix, T., Group, G.N.R., and Chesnut RM Videtta W, Temkin N, Dikmen S, Carney N, Petroni G, Lujan S, Pridgeon J, Barber J, Machamer J, Chaddock K, Celix J, Cherner M, Hendrix T, Alanis Mirones VS, La Fuente Zerain G, Lavadenz Cuentas A, Merida Maldonado R, Sandi Lora F, Jibaja Vega, R.C. (2012). A trial of intracranial-pressure monitoring in traumatic brain injury. N. Engl. J. Med. 367, 2471–2481.

26. Chiang, C.-C., Guo, S.-E., Huang, K.-C., Lee, B.-O., and Fan, J.-Y. (2016). Trajectories and associated factors of quality of life, global outcome, and post-concussion symptoms in the first year following mild traumatic brain injury. Qual. Life Res. 25, 2009–2019.

27. Cicuendez, M., Castano-Leon, A., Ramos, A., Hilario, A., Gomez, P.A., and Lagares, A. (2017). Prognostic value of corpus callosum injuries in severe head trauma. Acta Neurochir. (Wien). 159, 25–32.

28. Cipolle, M.D., Geffe, K., Getchell, J., Reed, J.F. 3rd, Fulda, G., Sugarman, M., and Tinkoff, G.H. (2014). Long-term outcome in elderly patients after operation for traumatic intracranial hemorrhage. Del. Med. J. 86, 237–244.

29. Cnossen, M.C., Polinder, S., Andriessen, T.M., Naalt, J., Haitsma, I., Horn, J., Franschman, G., Vos, P.E., Steyerberg, E.W., and Lingsma, H. (2017). Causes and Consequences of Treatment Variation in Moderate and Severe Traumatic Brain Injury: a Multicenter Study. Crit. Care Med. (no pagina.

30. Conley, Y.P., Okonkwo, D.O., Deslouches, S., Alexander, S., Puccio, A.M., Beers, S.R., and Ren, D. (2014). Mitochondrial polymorphisms impact outcomes after severe traumatic brain injury. J. Neurotrauma 31, 34–41.

31. Corral, L., Javierre, C.F., Ventura, J.L., Marcos, P., Herrero, J.I., and Manez, R. (2012). Impact of non-neurological complications in severe traumatic brain injury outcome. Crit. Care 16, R44.

32. Dahdah, M.N., Barisa, M.T., Schmidt, K., Barnes, S.A., Dubiel, R., Dunklin, C., Harper, C., Callender, L., Wilson, A., Diaz-Arrastia, R., and Shafi, S. (2014). Comparative effectiveness of traumatic brain injury rehabilitation: differential outcomes across TBI model systems centers. J. Head Trauma Rehabil. 29, 451–459.

33. Dahdah, M.N., Barnes, S., Buros, A., Dubiel, R., Dunklin, C., Callender, L., Harper, C., Wilson, A., Diaz-Arrastia, R., Bergquist, T., Sherer, M., Whiteneck, G., Pretz, C., Vanderploeg, R.D., and Shafi, S. (2016). Variations in Inpatient Rehabilitation Functional Outcomes Across Centers in the Traumatic Brain Injury Model Systems Study and the Influence of Demographics and Injury Severity on Patient Outcomes. Arch. Phys. Med. Rehabil. 97, 1821–1831.

34. Dams-O’Connor, K., Spielman, L., Singh, A., Gordon, W.A., Lingsma, H.F., Maas, A.I.R., Manley, G.T., Mukherjee, P., Okonkwo, D.O., Puccio, A.M., Schnyer, D.M., Valadka, A.B., Yue, J.K., Yuh, E.L., Casey, S.S., Cooper, S.R., Cheong, M., Hricik, A.J., Knight, E.E., Menon, D.K., Morabito, D.J., Pacheco, J.L., Sinha, T.K., and Vassar, M.J. (2013). The impact of previous traumatic brain injury on health and functioning: A TRACK-TBI study. J. Neurotrauma 30, 2014–2020.

35. Dams-O’Connor, K., Pretz, C., Billah, T., Hammond, F.M., and Harrison-Felix, C. (2015). Global Outcome Trajectories After TBI Among Survivors and Nonsurvivors: A National Institute on Disability and Rehabilitation Research Traumatic Brain Injury Model Systems Study. J. Head Trauma Rehabil. 30, E1-10.

36. Dardiotis, E., Paterakis, K., Tsivgoulis, G., Tsintou, M., Hadjigeorgiou, G.F., Dardioti, M., Grigoriadis, S., Simeonidou, C., Komnos, A., Kapsalaki, E., Fountas, K., and Hadjigeorgiou, G.M. (2014). AQP4 tag single nucleotide polymorphisms in patients with traumatic brain injury. J. Neurotrauma 31, 1920–1926.

37. S., de H., J.C., de G., B., J., and J., van der N. (2017). The association between microhaemorrhages and post - traumatic functional outcome in the chronic phase after mild traumatic brain injury. Neuroradiology 59, 963–969.

38. de Koning, M.E., Spikman, J.M., Coers, A., Schonherr, M.C., and van der Naalt, J. (2015). Pathways of care the first year after moderate and severe traumatic brain injury-discharge destinations and outpatient follow-up. Brain Inj. 29, 423–429.

39. Depreitere, B., Guiza, F., Van den Berghe, G., Schuhmann, M.U., Maier, G., Piper, I., and Meyfroidt, G. (2014). Pressure autoregulation monitoring and cerebral perfusion pressure target recommendation in patients with severe traumatic brain injury based on minute-by-minute monitoring data. J. Neurosurg. 120, 1451–1457.

40. Dhandapani, S.S., Manju, D., Sharma, B.S., and Mahapatra, A.K. (2012). Prognostic significance of age in traumatic brain injury. J. Neurosci. Rural Pract. 3, 131–135.

41. Dhandapani, S., Sarda, A.C., Kapoor, A., Salunke, P., Mathuriya, S.N., and Mukherjee, K.K. (2015). Validation of a New Clinico-Radiological Grading for Compound Head Injury: Implications on the Prognosis and the Need for Surgical Intervention. World Neurosurg. 84, 1244–1250.

42. Di Battista, A.P., Rhind, S.G., Hutchison, M.G., Hassan, S., Shiu, M.Y., Inaba, K., Topolovec-Vranic, J., Neto, A.C., Rizoli, S.B., and Baker, A.J. (2016). Inflammatory cytokine and chemokine profiles are associated with patient outcome and the hyperadrenergic state following acute brain injury. J. Neuroinflammation 13, 40.

43. Egea-Guerrero, J.J., Rodriguez-Rodriguez, A., Gordillo-Escobar, E., Fernandez-Delgado, E., Martinez-Roldan, A., Roldan-Reina, A., Duran-Martinez, P., de Vega-Rios, E., Freire-Aragon, M.D., Vilches-Arenas, A., Murillo-Cabezas, F., and Quintana-Diaz, M. (2017). IMPACT Score for Traumatic Brain Injury: Validation of the Prognostic Tool in a Spanish Cohort. J. Head Trauma Rehabil. .

44. Esnault, P., Nguyen, C., Bordes, J., D’Aranda, E., Montcriol, A., Contargyris, C., Cotte, J., Goutorbe, P., Joubert, C., Dagain, A., Boret, H., and Meaudre, E. (2017). Early-Onset Ventilator-Associated Pneumonia in Patients with Severe Traumatic Brain Injury: Incidence, Risk Factors, and Consequences in Cerebral Oxygenation and Outcome. Neurocrit. Care , 1–12.

45. Fabbri, A., Servadei, F., Marchesini, G., Bronzoni, C., Montesi, D., Arietta, L., Group, S.I. di M. d’Emergenza U.S., and Compagnoni MP Arici C, Belotti E, Arrigo C, Rosselli A, Fiorentino F, Gambilonghi F, Bertini L, Moscatelli P, Altomonte F, Cremonesi P, Zanna M, Mancini M, Pratesi M, Daghini E, Di Sarli M, Coen D, Pinelli G, Barozzi M, de Marinis GB, Allemand E, Pistoll, B.A. (2013). Antiplatelet therapy and the outcome of subjects with intracranial injury: the Italian SIMEU study. Crit. Care 17, R53.

46. Failla, M.D., Conley, Y.P., and Wagner, A.K. (2016). Brain-Derived Neurotrophic Factor (BDNF) in Traumatic Brain Injury-Related Mortality: Interrelationships Between Genetics and Acute Systemic and Central Nervous System BDNF Profiles. Neurorehabil. Neural Repair 30, 83–93.

47. Firsching, R., Woischneck, D., Langejurgen, A., Parreidt, A., Bondar, I., Skalej, M., Rohl, F., and Voellger, B. (2015). Clinical, Radiologic, and Legal Significance of “Extensor Response” in Posttraumatic Coma. J. Neurol. Surg. A. Cent. Eur. Neurosurg. 76, 456–465.

48. Forslund, M.V., Roe, C., Perrin, P.B., Sigurdardottir, S., Lu, J., Berntsen, S., and Andelic, N. (2017). The trajectories of overall disability in the first 5 years after moderate and severe traumatic brain injury. Brain Inj. 31, 329–335.

49. Galanaud, D., Perlbarg, V., Gupta, R., Stevens, R.D., Sanchez, P., Tollard, E., de Champfleur, N.M., Dinkel, J., Faivre, S., Soto-Ares, G., Veber, B., Cottenceau, V., Masson, F., Tourdias, T., André, E., Audibert, G., Schmitt, E., Ibarrola, D., Dailler, F., Vanhaudenhuyse, A., Tshibanda, L., Payen, J.-F., Le Bas, J.-F., Krainik, A., Bruder, N., Girard, N., Laureys, S., Benali, H., Puybasset, L., and Neuro Imaging for Coma Emergence and Recovery Consortium. (2012). Assessment of White Matter Injury and Outcome in Severe Brain Trauma. Anesthesiology 117, 1300–1310.

50. Gao, J.-B., Tang, W.-D., Wang, X., and Shen, J. (2014). Prognostic value of neuropeptide proenkephalin A in patients with severe traumatic brain injury. Peptides 58, 42–46.

51. Gao, L., Wu, X., Hu, J., Jin, Y., Han, X., Wu, X., Mao, Y., and Zhou, L. (2013). Intensive management and prognosis of 127 cases with traumatic bilateral frontal contusions. World Neurosurg. 80, 879–888.

52. Garner, A.A., Mann, K.P., Fearnside, M., Poynter, E., and Gebski, V. (2015). The Head Injury Retrieval Trial (HIRT): a single-centre randomised controlled trial of physician prehospital management of severe blunt head injury compared with management by paramedics only. Emerg. Med. J. 32, 869–875.

53. Garringer, J.A., Niyonkuru, C., McCullough, E.H., Loucks, T., Dixon, C.E., Conley, Y.P., Berga, S., and Wagner, A.K. (2013). Impact of aromatase genetic variation on hormone levels and global outcome after severe TBI. J. Neurotrauma 30, 1415–1425.

54. Godbolt, A.K., Stenberg, M., Jakobsson, J., Sorjonen, K., Krakau, K., Stalnacke, B.-M., and Nygren DeBoussard, C. (2015). Subacute complications during recovery from severe traumatic brain injury: frequency and associations with outcome. BMJ Open 5, e007208.

55. Goyal, A., Failla, M.D., Niyonkuru, C., Amin, K., Fabio, A., Berger, R.P., and Wagner, A.K. (2013). S100b as a prognostic biomarker in outcome prediction for patients with severe traumatic brain injury. J. Neurotrauma 30, 946–957.

56. Grauwmeijer, E., Heijenbrok-Kal, M.H., Haitsma, I.K., and Ribbers, G.M. (2012). A prospective study on employment outcome 3 years after moderate to severe traumatic brain injury. Arch. Phys. Med. Rehabil. 93, 993–999.

57. Gressot, L. V, Chamoun, R.B., Patel, A.J., Valadka, A.B., Suki, D., Robertson, C.S., and Gopinath, S.P. (2014). Predictors of outcome in civilians with gunshot wounds to the head upon presentation. J. Neurosurg. 121, 645–652.

58. Griesdale, D.E., Sekhon, M.S., Menon, D.K., Lavinio, A., Donnelly, J., Robba, C., Sekhon, I.S., Taylor, A., Henderson, W.R., Turgeon, A.F., and Gupta, A.K. (2015). Hemoglobin Area and Time Index Above 90 g/L are Associated with Improved 6-Month Functional Outcomes in Patients with Severe Traumatic Brain Injury. Neurocrit. Care 23, 78–84.

59. Guiza, F., Meyfroidt, G., Piper, I., Citerio, G., Chambers, I., Enblad, P., Nillson, P., Feyen, B., Jorens, P., Maas, A., Schuhmann, M.U., Donald, R., Moss, L., Van Den Berghe, G., and Depreitere, B. (2017). Cerebral Perfusion Pressure Insults and Associations with Outcome in Adult Traumatic Brain Injury. J. Neurotrauma 34, 2425–2431.

60. Guiza, F., Depreitere, B., Piper, I., Van den Berghe, G., and Meyfroidt, G. (2013). Novel methods to predict increased intracranial pressure during intensive care and long-term neurologic outcome after traumatic brain injury: development and validation in a multicenter dataset. Crit. Care Med. 41, 554–564.

61. Guo, C., Liu, L., Wang, B., and Wang, Z. (2017). Swirl sign in traumatic acute epidural hematoma: prognostic value and surgical management. Neurol. Sci. , 1–6.

62. Gupta, D., Sharma, D., Kannan, N., Prapruettham, S., Mock, C., Wang, J., Qiu, Q., Pandey, R.M., Mahapatra, A., Dash, H.H., Hecker, J.G., Rivara, F.P., Rowhani-Rahbar, A., and Vavilala, M.S. (2016). Guideline Adherence and Outcomes in Severe Adult Traumatic Brain Injury for the CHIRAG (Collaborative Head Injury and Guidelines) Study. World Neurosurg. 89, 169–179.

63. Haagsma, J.A., Scholten, A.C., Andriessen, T.M.J.C., Vos, P.E., Van Beeck, E.F., and Polinder, S. (2015). Impact of depression and post-traumatic stress disorder on functional outcome and health-related quality of life of patients with mild traumatic brain injury. J. Neurotrauma 32, 853–862.

64. Hamed, M., Schuss, P., Daher, F.H., Borger, V., Guresir, A., Vatter, H., and Guresir, E. (2016). Acute Traumatic Subdural Hematoma: Surgical Management in the Presence of Cerebral Herniation-A Single-Center Series and Multivariate Analysis. World Neurosurg. 94, 501–506.

65. Han, J., King, N.K.K., Neilson, S.J., Gandhi, M.P., and Ng, I. (2014). External validation of the CRASH and IMPACT prognostic models in severe traumatic brain injury. J. Neurotrauma 31, 1146–1152.

66. Harrison, D., Prabhu, G., Grieve, R., Harvey, S., Sadique, M., Gomes, M., Griggs, K., Walmsley, E., Smith, M., Yeoman, P., Lecky, F., Hutchinson, P., Menon, D., and Rowan, K. (2013). Risk Adjustment In Neurocritical care (RAIN) – prospective validation of risk prediction models for adult patients with acute traumatic brain injury to use to evaluate the optimum location and comparative costs of neurocritical care: a cohort study. Heal. Technol Assess 17, vii–viii, 1-350.

67. Hart, T., Whyte, J., Poulsen, I., Kristensen, K.S., Nordenbo, A.M., Chervoneva, I., and Vaccaro, M.J. (2016). How Do Intensity and Duration of Rehabilitation Services Affect Outcomes From Severe Traumatic Brain Injury? A Natural Experiment Comparing Health Care Delivery Systems in 2 Developed Nations. Arch. Phys. Med. Rehabil. 97, 2045–2053.

68. Hatefi, M., Dastjerdi, M.M., Ghiasi, B., and Rahmani, A. (2016). Association of serum uric acid level with the severity of brain injury and patient’s outcome in severe traumatic brain injury. J. Clin. Diagnostic Res. 10, OC20-OC24.

69. Hellstrom, T., Kaufmann, T., Andelic, N., Soberg, H.L., Sigurdardottir, S., Helseth, E., Andreassen, O.A., and Westlye, L.T. (2017). Predicting outcome 12 months after mild traumatic brain injury in patients admitted to a neurosurgery service. Front. Neurol. 8, 125.

70. Henninger, N., Izzy, S., Carandang, R., Hall, W., and Muehlschlegel, S. (2014). Severe leukoaraiosis portends a poor outcome after traumatic brain injury. Neurocrit. Care 21, 483–495.

71. Herrera-Melero, M.C., Egea-Guerrero, J.J., Vilches-Arenas, A., Rincon-Ferrari, M.D., Flores-Cordero, J.M., Leon-Carrion, J., and Murillo-Cabezas, F. (2015). Acute predictors for mortality after severe TBI in Spain: Gender differences and clinical data. Brain Inj. 29, 1439–1444.

72. Honeybul, S., and Ho, K.M. (2016). Predicting long-term neurological outcomes after severe traumatic brain injury requiring decompressive craniectomy: A comparison of the CRASH and IMPACT prognostic models. Injury 47, 1886–1892.

73. Hua, L., Wenming, W., Feng, C., Qiang, Y., Jian, Y., Jin, H., and Ren, G. (2014). External ventricular drains versus intraparenchymal intracranial pressure monitors in traumatic brain injury: A prospective observational study. World Neurosurg. 83, 794–800.

74. Huang, Y.-H., Lee, T.-C., Yang, K.-Y., and Liao, C.-C. (2013). Is timing of cranioplasty following posttraumatic craniectomy related to neurological outcome?. Int. J. Surg. 11, 886–890.

75. Hudak, A.M., Hynan, L.S., Harper, C.R., and Diaz-Arrastia, R. (2012). Association of depressive symptoms with functional outcome after traumatic brain injury. J. Head Trauma Rehabil. 27, 87–98.

76. Hutchinson, P.J., Kolias, A.G., Timofeev, I.S., Corteen, E.A., Czosnyka, M., Timothy, J., Anderson, I., Bulters, D.O., Belli, A., Eynon, C.A., Wadley, J., Mendelow, A.D., Mitchell, P.M., Wilson, M.H., Critchley, G., Sahuquillo, J., Unterberg, A., Servadei, F., Teasdale, G.M., Pickard, J.D., Menon, D.K., Murray, G.D., Kirkpatrick, P.J., Collaborators, Rescue.T., and Bell BA Latronico N, Moody C, Rickels E, Shaw DM, Smith M, Richards H, Maas AI, Menon DK, Stocchetti N, Teasdale GM, Hutchinson, Kirkpatrick PJ, Timofeev I, Kolias AG, Adams H, Li LM, Deakin N, Fazekas B, Corteen EA, Grainger S, Melhorn J, Ingham S, Hora, D.N.M. (2016). Trial of Decompressive Craniectomy for Traumatic Intracranial Hypertension. N. Engl. J. Med. 375, 1119–1130.

77. Iaccarino, C., Schiavi, P., Picetti, E., Goldoni, M., Cerasti, D., Caspani, M., and Servadei, F. (2014). Patients with brain contusions: predictors of outcome and relationship between radiological and clinical evolution. J. Neurosurg. 120, 908–918.

78. Jacobs, B., Beems, T., van der Vliet, T.M., van Vugt, A.B., Hoedemaekers, C., Horn, J., Franschman, G., Haitsma, I., van der Naalt, J., Andriessen, T.M.J.C., Borm, G.F., and Vos, P.E. (2013). Outcome prediction in moderate and severe traumatic brain injury: a focus on computed tomography variables. Neurocrit. Care 19, 79–89.

79. Jin, Y., Li, B.-Y., Qiu, L.-L., Ling, Y.-R., and Bai, Z.-Q. (2012). Decreased plasma gelsolin is associated with 1-year outcome in patients with traumatic brain injury. J. Crit. Care 27, 527.e1-6.

80. Junaid, M., Mamoon-ur-Rashid, Afsheen, A., Tahir, A., Bukhari, S.S., and Kalsoom, A. (2016). Changing spectrum of traumatic head injuries: Demographics and outcome analysis in a tertiary care referral center. J. Pak. Med. Assoc. 66, 864–868.

81. Kaloostian, P., Robertson, C., Gopinath, S.P., Stippler, M., King, C.C., Qualls, C., Yonas, H., and Nemoto, E.M. (2012). Outcome prediction within twelve hours after severe traumatic brain injury by quantitative cerebral blood flow. J. Neurotrauma 29, 727–734.

82. Kasprowicz, M., Burzynska, M., Melcer, T., and Kubler, A. (2016). A comparison of the Full Outline of UnResponsiveness (FOUR) score and Glasgow Coma Score (GCS) in predictive modelling in traumatic brain injury. Br. J. Neurosurg. 30, 211–220.

83. Katsnelson, M., Mackenzie, L., Frangos, S., Oddo, M., Levine, J.M., Pukenas, B., Faerber, J., Dong, C., Kofke, W.A., and le Roux, P.D. (2012). Are initial radiographic and clinical scales associated with subsequent intracranial pressure and brain oxygen levels after severe traumatic brain injury?. Neurosurgery 70, 1095–1105.

84. Kesinger, M.R., Kumar, R.G., Wagner, A.K., Puyana, J.C., Peitzman, A.P., Billiar, T.R., and Sperry, J.L. (2015). Hospital-acquired pneumonia is an independent predictor of poor global outcome in severe traumatic brain injury up to 5 years after discharge. J. Trauma Acute Care Surg. 78, 396–402.

85. Khalili, H., Niakan, A., Ghaffarpasand, F., Kiani, A., and Behjat, R. (2017). Outcome determinants of decompressive craniectomy in patients with traumatic brain injury; A single center experience from Southern Iran. Bull. Emerg. Trauma 5, 190–196.

86. Khalili, H., Niakan, A., and Ghaffarpasand, F. (2017). Effects of cerebrolysin on functional recovery in patients with severe disability after traumatic brain injury: A historical cohort study. Clin. Neurol. Neurosurg. 152, 34–38.

87. Khalili, H., Sadraei, N., Niakan, A., Ghaffarpasand, F., and Sadraei, A. (2016). Role of Intracranial Pressure Monitoring in Management of Patients with Severe Traumatic Brain Injury: Results of a Large Level I Trauma Center in Southern Iran. World Neurosurg. 94, 120–125.

88. H.J., K. (2012). The prognostic factors related to traumatic brain stem injury. J. Korean Neurosurg. Soc. 51, 24–30.

89. Korley, F.K., Diaz-Arrastia, R., Wu, A.H.B., Yue, J.K., Manley, G.T., Sair, H.I., Van Eyk, J., Everett, A.D., investigators, T.-T., Okonkwo, D.O., Valadka, A.B., Gordon, W.A., Maas, A.I.R., Mukherjee, P., Yuh, E.L., Lingsma, H.F., Puccio, A.M., and Schnyer, D.M. (2016). Circulating Brain-Derived Neurotrophic Factor Has Diagnostic and Prognostic Value in Traumatic Brain Injury. J. Neurotrauma 33, 215–225.

90. Kumar, R.G., Diamond, M.L., Boles, J.A., Berger, R.P., Tisherman, S.A., Kochanek, P.M., and Wagner, A.K. (2015). Acute CSF interleukin-6 trajectories after TBI: associations with neuroinflammation, polytrauma, and outcome. Brain. Behav. Immun. 45, 253–262.

91. Leal-Noval, S.R., Munoz-Serrano, A., Arellano-Orden, V., Cayuela, A., Munoz-Gomez, M., Recio, A., Alcantara, A., Amaya-Villar, R., Casado-Mendez, M., and Murillo-Cabezas, F. (2016). Effects of Red Blood Cell Transfusion on Long-Term Disability of Patients with Traumatic Brain Injury. Neurocrit. Care 24, 371–380.

92. Lecky, F.E., Russell, W., McClelland, G., Pennington, E., Fuller, G., Goodacre, S., Han, K., Curran, A., Holliman, D., Chapman, N., Freeman, J., Byers, S., Mason, S., Potter, H., Coats, T., Mackway-Jones, K., Peters, M., and Shewan, J. (2017). Bypassing nearest hospital for more distant neuroscience care in head-injured adults with suspected traumatic brain injury: Findings of the head injury transportation straight to neurosurgery (HITS-NS) pilot cluster randomised trial. BMJ Open 7, e016355.

93. Leitgeb, J., Mauritz, W., Brazinova, A., Majdan, M., and Wilbacher, I. (2013). Impact of concomitant injuries on outcomes after traumatic brain injury. Arch. Orthop. Trauma Surg. 133, 659–668.

94. Leitgeb, J., Mauritz, W., Brazinova, A., Matula, C., Majdan, M., Wilbacher, I., and Rusnak, M. (2012). Outcome of patients with severe brain trauma who were treated either by neurosurgeons or by trauma surgeons. J. Trauma Acute Care Surg. 72, 1263–1270.

95. Lewis, P.M., Smielewski, P., Rosenfeld, J. V, Pickard, J.D., and Czosnyka, M. (2012). Monitoring of the association between cerebral blood flow velocity and intracranial pressure. Acta Neurochir. Suppl. 114, 147–151.

96. Z.-M., L., L.-X., W., L.-C., J., J.-X., Z., F.-Y., G., and F., Q. (2012). Surgical treatment of transtentorial herniation after traumatic brain injury. Neurosurg. Q. 22, 26–29.

97. Z., L., Z., Q., N., Z., J., Z., and D., S. (2016). Comparison between intraventricular and intraparenchymal intracranial pressure monitoring in Asian patients with severe traumatic brain injury. Neurosurg. Q. 26, 120–124.

98. Li, Z.-M., Xiao, Y.-L., Zhu, J.-X., Geng, F.-Y., Guo, C.-J., Chong, Z.-L., and Wang, L.-X. (2016). Recombinant human erythropoietin improves functional recovery in patients with severe traumatic brain injury: A randomized, double blind and controlled clinical trial. Clin. Neurol. Neurosurg. 150, 80–83.

99. Limpastan, K., Norasetthada, T., Watcharasaksilp, W., and Vaniyapong, T. (2013). Factors influencing the outcome of decompressive craniectomy used in the treatment of severe traumatic brain injury. J. Med. Assoc. Thai. 96, 678–682.

100. Lu, H.-Y., Li, T.-C., Tu, Y.-K., Tsai, J.-C., Lai, H.-S., and Kuo, L.-T. (2015). Predicting long-term outcome after traumatic brain injury using repeated measurements of Glasgow Coma Scale and data mining methods. J. Med. Syst. 39, 14.

101. Lupi, A., Bertagnoni, G., Borghero, A., Picelli, A., Cuccurullo, V., and Zanco, P. (2014). 18FDG-PET/CT in traumatic brain injury patients: the relative hypermetabolism of vermis cerebelli as a medium and long term predictor of outcome. Curr. Radiopharm. 7, 57–62.

102. Maekawa, T., Yamashita, S., Nagao, S., Hayashi, N., Ohashi, Y., Group, B.-H.S., and Aibiki M Asai Y, Dohi K, Eguchi Y, Fujita M, Fukuoka T, Ikeda K, Iwashita T, Kaneda K, Kaneko T, Kato Y, Kawakita K, Kinoshita K, Kitahara T, Kitazawa K, Kobata H, Koizumi H, Kuroda Y, Marukawa S, Mori K, Nakamura H, Nakamura S, Nakatsukasa M, Ninomiya N, A.T. (2015). Prolonged mild therapeutic hypothermia versus fever control with tight hemodynamic monitoring and slow rewarming in patients with severe traumatic brain injury: a randomized controlled trial. J. Neurotrauma 32, 422–429.

103. Majdan, M., Brazinova, A., Wilbacher, I., Rusnak, M., and Mauritz, W. (2015). The impact of body mass index on severity, patterns and outcomes after traumatic brain injuries caused by low level falls. Eur. J. Trauma Emerg. Surg. 41, 651–656.

104. Majdan, M., Lingsma, H.F., Nieboer, D., Mauritz, W., Rusnak, M., and Steyerberg, E.W. (2014). Performance of IMPACT, CRASH and Nijmegen models in predicting six month outcome of patients with severe or moderate TBI: an external validation study. Scand. J. Trauma. Resusc. Emerg. Med. 22, 68.

105. Matsukawa, H., Shinoda, M., Fujii, M., Takahashi, O., Murakata, A., and Yamamoto, D. (2013). Acute alcohol intoxication, diffuse axonal injury and intraventricular bleeding in patients with isolated blunt traumatic brain injury. Brain Inj. 27, 1409–1414.

106. Matsukawa, H., Shinoda, M., Fujii, M., Takahashi, O., Murakata, A., Yamamoto, D., Sumiyoshi, S., and Ishikawa, R. (2012). Intraventricular hemorrhage on computed tomography and corpus callosum injury on magnetic resonance imaging in patients with isolated blunt traumatic brain injury. J. Neurosurg. 117, 334–339.

107. Matsushima, K., Peng, M., Velasco, C., Schaefer, E., Diaz-Arrastia, R., and Frankel, H. (2012). Glucose variability negatively impacts long-term functional outcome in patients with traumatic brain injury. J. Crit. Care 27, 125–131.

108. Mauritz, W., Brazinova, A., Majdan, M., Rehorcikova, V., and Leitgeb, J. (2014). Effects of time of hospital admission on outcomes after severe traumatic brain injury in Austria. Wien. Klin. Wochenschr. 126, 278–285.

109. McNett, M.M., Amato, S., and Philippbar, S.A. (2016). A Comparative Study of Glasgow Coma Scale and Full Outline of Unresponsiveness Scores for Predicting Long-Term Outcome After Brain Injury. J. Neurosci. Nurs. 48, 207–214.

110. Mendelow, A.D., Gregson, B.A., Rowan, E.N., Francis, R., McColl, E., McNamee, P., Chambers, I.R., Unterberg, A., Boyers, D., Mitchell, P.M., Investigators, S., and Mendelow A Mitchell PM, Unterberg A, McColl EM, Chambers IR, McNamee P, Steers J, Vail A, Birchall D, Timothy J, Vale L, White A, O’Meara D, Mendelow AD, Gregson BA, Mitchell PM, Unterberg A, McColl EM, Chambers IR, McNamee P, Rowan EN, Boyers D, Francis, G.B.A. (2015). Early Surgery versus Initial Conservative Treatment in Patients with Traumatic Intracerebral Hemorrhage (STITCH[Trauma]): The First Randomized Trial. J. Neurotrauma 32, 1312–1323.

111. Merzo, A., Lenell, S., Nyholm, L., Enblad, P., and Lewen, A. (2016). Promising clinical outcome of elderly with TBI after modern neurointensive care. Acta Neurochir. (Wien). 158, 125–133.

112. Moen, K.G., Brezova, V., Skandsen, T., Haberg, A.K., Folvik, M., and Vik, A. (2014). Traumatic axonal injury: the prognostic value of lesion load in corpus callosum, brain stem, and thalamus in different magnetic resonance imaging sequences. J. Neurotrauma 31, 1486–1496.

113. Munivenkatappa, A., Devi, B.I., Gregor, T.I., Bhat, D.I., Kumarsamy, A.D., and Shukla, D.P. (2013). Bicycle accident-related head injuries in India. J. Neurosci. Rural Pract. 4, 262–266.

114. Nelson, D.W., Rudehill, A., MacCallum, R.M., Holst, A., Wanecek, M., Weitzberg, E., and Bellander, B.-M. (2012). Multivariate outcome prediction in traumatic brain injury with focus on laboratory values. J. Neurotrauma 29, 2613–2624.

115. NICE-SUGAR Study Investigators for the Australian and New Zealand Intensive Care Society Clinical Trials Group and the Canadian Critical Care Trials Group, Finfer, S., Chittock, D., Li, Y., Foster, D., Dhingra, V., Bellomo, R., Cook, D., Dodek, P., Hebert, P., Henderson, W., Heyland, D., Higgins, A., McArthur, C., Mitchell, I., Myburgh, J., Robinson, B., Ronco, J., and Finfer S Bellomo R, McArthur C, Mitchell I, Myburgh J, Norton R, Potter J, Chittock D, Dhingra V, Foster D, Cook D, Dodek P, Hebert P, Henderson W, Heyland D, McDonald E, Ronco J, Schweitzer I, Peto R, Sandercock P, Sprung C, Young J, Li Y, Li Q, Bompoin, B.D. (2015). Intensive versus conventional glucose control in critically ill patients with traumatic brain injury: long-term follow-up of a subgroup of patients from the NICE-SUGAR study. Intensive Care Med. 41, 1037–1047.

116. Nichol, A., French, C., Little, L., Haddad, S., Presneill, J., Arabi, Y., Bailey, M., Cooper, D.J., Duranteau, J., Huet, O., Mak, A., McArthur, C., Pettila, V., Skrifvars, M., Vallance, S., Varma, D., Wills, J., Bellomo, R., Investigators, E.-T., Group, A.C.T., and Bellomo R French C, Cooper D, Huet O, Little L, Mak A, Pettila V, Presneill J, Vallance S, Varma D, Wills J, Duranteau J, Vicaut E, Gallula P, Raghavan V, Chamam A, Kambire S, Camelo S, Cook D, Crowther M, Brun-Buisson C, Lauzier F, Howe B, Paul E, Baile, N.A. (2015). Erythropoietin in traumatic brain injury (EPO-TBI): a double-blind randomised controlled trial. Lancet (London, England) 386, 2499–2506.

117. Nishijima, D.K., Melnikow, J., Tancredi, D.J., Shahlaie, K., Utter, G.H., Galante, J.M., Rudisill, N., and Holmes, J.F. (2015). Long-term neurological outcomes in adults with traumatic intracranial hemorrhage admitted to ICU versus floor. West. J. Emerg. Med. 16, 284–290.

118. Osier, N.D., Bales, J.W., Pugh, B., Shin, S., Wyrobek, J., Puccio, A.M., Okonkwo, D.O., Ren, D., Alexander, S., Conley, Y.P., and Dixon, C.E. (2017). Variation in PPP3CC genotype is associated with long-term recovery after severe brain injury. J. Neurotrauma 34, 86–96.

119. Ostberg, A., and Tenovuo, O. (2014). Smoking and outcome of traumatic brain injury. Brain Inj. 28, 155–160.

120. Ozyurt, E., Goksu, E., Cengiz, M., Yilmaz, M., and Ramazanoglu, A. (2015). Retrospective Analysis of Prognostic Factors of Severe Traumatic Brain Injury in a University Hospital in Turkey. Turk. Neurosurg. 25, 877–882.

121. Pakkanen, T., Virkkunen, I., Kamarainen, A., Huhtala, H., Silfvast, T., Virta, J., Randell, T., and Yli-Hankala, A. (2016). Pre-hospital severe traumatic brain injury - comparison of outcome in paramedic versus physician staffed emergency medical services. Scand. J. Trauma. Resusc. Emerg. Med. 24, 62.

122. Pan, J.-W., Gao, X.-W., Jiang, H., Li, Y.-F., Xiao, F., and Zhan, R.-Y. (2015). Low serum ficolin-3 levels are associated with severity and poor outcome in traumatic brain injury. J. Neuroinflammation 12, 226.

123. Panczykowski, D.M., Puccio, A.M., Scruggs, B.J., Bauer, J.S., Hricik, A.J., Beers, S.R., and Okonkwo, D.O. (2012). Prospective Independent Validation of IMPACT Modeling as a Prognostic Tool in Severe Traumatic Brain Injury. J. Neurotrauma 29, 47–52.

124. Patel, M.B., Wilson, L.D., Bregman, J.A., Leath, T.C., Humble, S.S., Davidson, M.A., de Riesthal, M.R., and Guillamondegui, O.D. (2015). Neurologic Functional and Quality of Life Outcomes after TBI: Clinic Attendees versus Non-Attendees. J. Neurotrauma 32, 984–989.

125. Ponce, L.L., Pillai, S., Cruz, J., Li, X., Julia, H., Gopinath, S., and Robertson, C.S. (2012). Position of probe determines prognostic information of brain tissue PO2 in severe traumatic brain injury. Neurosurgery 70, 1492–1493.

126. Prathep, S., Sriplung, H., Phuenpathom, N., Zunt, J., Hirunpat, S., and Vavilala, M.S. (2017). Characteristics and outcomes of thai patients hospitalized with severe traumatic brain injury between 2009 and 2011. J. Med. Assoc. Thail. 100, 843–849.

127. Prieto-Palomino, M.A., Curiel-Balsera, E., Arias-Verdu, M.D., Der Kroft, M.D.-V., Munoz-Lopez, A., Fernandez-Ortega, J.F., Quesada-Garcia, G., Sanchez-Cantalejo, E., and Rivera-Fernandez, R. (2016). Relationship between quality-of-life after 1-year follow-up and severity of traumatic brain injury assessed by computerized tomography. Brain Inj. 30, 441–451.

128. Qiu, B., Xu, S., Fang, L., Chotai, S., Li, W., and Qi, S. (2012). Surgical strategies for neurological function preservation in severe brain contusion. Turk. Neurosurg. 22, 329–335.

129. Rahmani, A., Hatefi, M., Dastjerdi, M.M., Zare, M., Imani, A., and Shirazi, D. (2016). Correlation Between Serum Homocysteine Levels and Outcome of Patients with Severe Traumatic Brain Injury. World Neurosurg. 87, 507–515.

130. Raj, R., Siironen, J., Kivisaari, R., Hernesniemi, J., and Skrifvars, M.B. (2014). Predicting outcome after traumatic brain injury: development of prognostic scores based on the IMPACT and the APACHE II. J. Neurotrauma 31, 1721–1732.

131. Rizoli, S.B., Jaja, B.N.R., Di Battista, A.P., Rhind, S.G., Neto, A.C., da Costa, L., Inaba, K., da Luz, L.T., Nascimento, B., A., P., Baker, A.J., and de Oliveira Manoel, A.L. (2017). Catecholamines as outcome markers in isolated traumatic brain injury: The COMA-TBI study. Crit. Care 21, 37.

132. Robertson, C.S., Hannay, H.J., Yamal, J.-M., Gopinath, S., Goodman, J.C., Tilley, B.C., Investigators, E.S.T.B.I.T., Baldwin, A., Rivera Lara, L., Saucedo-Crespo, H., Ahmed, O., Sadasivan, S., Ponce, L., Cruz-Navarro, J., Shahin, H., Aisiku, I.P., Doshi, P., Valadka, A., Neipert, L., Waguspack, J.M., Rubin, M.L., Benoit, J.S., and Swank, P. (2014). Effect of erythropoietin and transfusion threshold on neurological recovery after traumatic brain injury: a randomized clinical trial. JAMA 312, 36–47.

133. Roozenbeek, B., Lingsma, H.F., Lecky, F.E., Lu, J., Weir, J., Butcher, I., McHugh, G.S., Murray, G.D., Perel, P., Maas, A.I., Steyerberg, E.W., Group, I.M. on P.A. of C.T. in T.B.I. (IMPACT) S., Collaborators, C.R.A.S.H.I. (CRASH) T., and (TARN), T.A. and R.N. (2012). Prediction of outcome after moderate and severe traumatic brain injury: external validation of the International Mission on Prognosis and Analysis of Clinical Trials (IMPACT) and Corticoid Randomisation After Significant Head injury (CRASH) prognostic mod. Crit. Care Med. 40, 1609–1617.

134. Rundhaug, N.P., Moen, K.G., Skandsen, T., Schirmer-Mikalsen, K., Lund, S.B., Hara, S., and Vik, A. (2015). Moderate and severe traumatic brain injury: effect of blood alcohol concentration on Glasgow Coma Scale score and relation to computed tomography findings. J. Neurosurg. 122, 211–218.

135. Santarsieri, M., Niyonkuru, C., McCullough, E.H., Dobos, J.A., Dixon, C.E., Berga, S.L., and Wagner, A.K. (2014). Cerebrospinal fluid cortisol and progesterone profiles and outcomes prognostication after severe traumatic brain injury. J. Neurotrauma 31, 699–712.

136. Scheenen, M.E., de Koning, M.E., van der Horn, H.J., Roks, G., Yilmaz, T., van der Naalt, J., and Spikman, J.M. (2016). Acute Alcohol Intoxication in Patients with Mild Traumatic Brain Injury: Characteristics, Recovery, and Outcome. J. Neurotrauma 33, 339–345.

137. Scholten, A.C., Haagsma, J.A., Andriessen, T.M.J.C., Vos, P.E., Steyerberg, E.W., van Beeck, E.F., and Polinder, S. (2015). Health-related quality of life after mild, moderate and severe traumatic brain injury: patterns and predictors of suboptimal functioning during the first year after injury. Injury 46, 616–624.

138. Sekhon, M.S., Gooderham, P., Toyota, B., Kherzi, N., Hu, V., Dhingra, V.K., Hameed, M.S., Chittock, D.R., and Griesdale, D.E. (2017). Implementation of Neurocritical Care Is Associated with Improved Outcomes in Traumatic Brain Injury. Can. J. Neurol. Sci. 44, 350–357.

139. Sherif, C., Di Ieva, A., Gibson, D., Pakrah-Bodingbauer, B., Widhalm, G., Krusche-Mandl, I., Erdoes, J., Gilloon, B., and Matula, C. (2012). A management algorithm for cerebrospinal fluid leak associated with anterior skull base fractures: detailed clinical and radiological follow-up. Neurosurg. Rev. 35, 227–228.

140. Shi, L., Sun, G., Qian, C., Pan, T., Li, X., Zhang, S., and Wang, Z. (2015). Technique of stepwise intracranial decompression combined with external ventricular drainage catheters improves the prognosis of acute post-traumatic cerebral hemispheric brain swelling patients. Front. Hum. Neurosci. 9, 535.

141. Shimoda, K., Maeda, T., Tado, M., Yoshino, A., Katayama, Y., and Bullock, M.R. (2014). Outcome and surgical management for geriatric traumatic brain injury: analysis of 888 cases registered in the Japan Neurotrauma Data Bank. World Neurosurg. 82, 1300–1306.

142. Siddiqui, U., Tahir, M., Shamim, M., and Enam, S. (2015). Clinical outcome and cost effectiveness of early tracheostomy in isolated severe head injury patients. Surg. Neurol. Int. 6, 65.

143. Sigurdardottir, S., Andelic, N., Skandsen, T., Anke, A., Roe, C., Holthe, O.O., and Wehling, E. (2016). Olfactory identification and its relationship to executive functions, memory, and disability one year after severe traumatic brain injury. Neuropsychology 30, 98–108.

144. Sinha, S., Raheja, A., Garg, M., Moorthy, S., Agrawal, D., Gupta, D.K., Satyarthee, G.D., Singh, P.K., Borkar, S.A., Gurjar, H., V., T., R.M., P., and B.S., S. (2015). Decompressive craniectomy in traumatic brain injury: A single-center, multivariate analysis of 1,236 patients at a tertiary care hospital in India. Neurol. India 63, 175–183.

145. Skolnick, B.E., Maas, A.I., Narayan, R.K., van der Hoop, R.G., MacAllister, T., Ward, J.D., Nelson, N.R., Stocchetti, N., Investigators, S.T., and Marmarou A Narayan R, Skolnick BE, Ward J, Stocchetti N, Dearden NM, Clarence-Smith K, Genazzani AR, Grady MS, Steyerberg EW, Okonkwo D, Grieve G, Zaaroor M, Levi L, Smrcka M, May A, Pachl J, Manji M, Chen J, Pichon N, Lobato RD, Norasetthada T, Puybasse, M.A.I. (2014). A clinical trial of progesterone for severe traumatic brain injury. N. Engl. J. Med. 371, 2467–2476.

146. Soberg, H.L., Roe, C., Anke, A., Arango-Lasprilla, J.C., Skandsen, T., Sveen, U., von Steinbuchel, N., and Andelic, N. (2013). Health-related quality of life 12 months after severe traumatic brain injury: a prospective nationwide cohort study. J. Rehabil. Med. 45, 785–791.

147. Stein, D.M., Brenner, M., Hu, P.F., Yang, S., Hall, E.C., Stansbury, L.G., Menaker, J., and Scalea, T.M. (2013). Timing of intracranial hypertension following severe traumatic brain injury. Neurocrit. Care 18, 332–340.

148. Stocchetti, N., Paterno, R., Citerio, G., Beretta, L., and Colombo, A. (2012). Traumatic brain injury in an aging population. J. Neurotrauma 29, 1119–1125.

149. Sun, G., Shi, L., Pan, T., Li, X., and Zhang, S. (2016). Technique of ICP monitored stepwise intracranial decompression effectively reduces postoperative complications of severe bifrontal contusion. Front. Neurol. 7, 56.

150. Sveen, U., Andelic, N., Bautz-Holter, E., and Roe, C. (2015). Self-reported competency--validation of the Norwegian version of the patient competency rating scale for traumatic brain injury. Disabil. Rehabil. 37, 239–246.

151. Takala, R.S.K., Posti, J.P., Runtti, H., Newcombe, V.F., Outtrim, J., Katila, A.J., Frantzén, J., Ala-Seppälä, H., Kyllönen, A., Maanpää, H.-R., Tallus, J., Hossain, M.I., Coles, J.P., Hutchinson, P., van Gils, M., Menon, D.K., and Tenovuo, O. (2016). Glial Fibrillary Acidic Protein and Ubiquitin C-Terminal Hydrolase-L1 as Outcome Predictors in Traumatic Brain Injury. World Neurosurg. 87, 8–20.

152. Taw, B.B.T., Lam, A.C.S., Ho, F.L.Y., Hung, K.N., Lui, W.M., and Leung, G.K.K. (2012). Functional survival after acute care for severe head injury at a designated trauma center in Hong Kong. Asian J. Surg. 35, 117–122.

153. Theadom, A., Parmar, P., Jones, K., Barker-Collo, S., Starkey, N.J., McPherson, K.M., Ameratunga, S., Feigin, V.L., and Group, B.R. (2015). Frequency and impact of recurrent traumatic brain injury in a population-based sample. J. Neurotrauma 32, 674–681.

154. Thelin, E.P., Nelson, D.W., and Bellander, B.-M. (2014). Secondary peaks of S100B in serum relate to subsequent radiological pathology in traumatic brain injury. Neurocrit. Care 20, 217–229.

155. Thelin, E.P., Nelson, D.W., Vehviläinen, J., Nyström, H., Kivisaari, R., Siironen, J., Svensson, M., Skrifvars, M.B., Bellander, B.-M., and Raj, R. (2017). Evaluation of novel computerized tomography scoring systems in human traumatic brain injury: An observational, multicenter study. PLOS Med. 14, e1002368.

156. van der Horn, H.J., Spikman, J.M., Jacobs, B., and van der Naalt, J. (2013). Postconcussive complaints, anxiety, and depression related to vocational outcome in minor to severe traumatic brain injury. Arch. Phys. Med. Rehabil. 94, 867–874.

157. van der Naalt, J., Timmerman, M.E., de Koning, M.E., van der Horn, H.J., Scheenen, M.E., Jacobs, B., Hageman, G., Yilmaz, T., Roks, G., and Spikman, J.M. (2017). Early predictors of outcome after mild traumatic brain injury (UPFRONT): an observational cohort study. Lancet Neurol. 16, 532–540.

158. Vathanalaoha, K., Oearsakul, T., and Tunthanathip, T. (2017). Predictive factors of survival and 6-month favorable outcome of very severe head trauma patients; a historical cohort study. Emergency 5, 128–136.

159. Wagner, A.K., Hatz, L.E., Scanlon, J.M., Niyonkuru, C., Miller, M.A., Ricker, J.H., Conley, Y.P., and Ferrell, R.E. (2012). Association of KIBRA rs17070145 polymorphism and episodic memory in individuals with severe TBI. Brain Inj. 26, 1658–1669.

160. Walker, W.C., Ketchum, J.S. 3rd, Marwitz, J.H., Kolakowsky-Hayner, S.A., McClish, D.K., and Bushnik, T. (2015). Global Outcome and Late Seizures After Penetrating Versus Closed Traumatic Brain Injury: A NIDRR TBI Model Systems Study. J. Head Trauma Rehabil. 30, 231–240.

161. Wan, X., Zhao, K., Wang, S., Zhang, H., Zeng, L., Wang, Y., Han, L., Beejadhursing, R., Shu, K., and Lei, T. (2017). Is It Reliable to Predict the Outcome of Elderly Patients with Severe Traumatic Brain Injury Using the IMPACT Prognostic Calculator?. World Neurosurg. 103, 584–590.

162. Wang, W., Hu, L., Lin, H., Li, J., Luo, F., Huang, W., Lin, J., Cai, G., and Liu, C. (2014). Risk factors for post-traumatic massive cerebral infarction secondary to space-occupying epidural hematoma. J. Neurotrauma 31, 1444–1450.

163. Wang, Z.L., Xu, D.S., Wang, Y.X., Qin, H., and Geng, D. (2015). Effect of single nucleotide polymorphisms in the ATP-binding cassette B1 gene on the clinical outcome of traumatic brain injury. Genet. Mol. Res. 14, 10948–10953.

164. Waqas, M., Bakhshi, S.K., Shamim, M.S., and Anwar, S. (2016). Radiological prognostication in patients with head trauma requiring decompressive craniectomy: Analysis of optic nerve sheath diameter and Rotterdam CT Scoring System. J. Neuroradiol. 43, 25–30.

165. Waqas, M., Shamim, M.S., Enam, S.F., Qadeer, M., Bakhshi, S.K., Patoli, I., and Ahmad, K. (2016). Predicting outcomes of decompressive craniectomy: use of Rotterdam Computed Tomography Classification and Marshall Classification. Br. J. Neurosurg. 30, 258–263.

166. Waters, R.J., Murray, G.D., Teasdale, G.M., Stewart, J., Day, I., Lee, R.J., and Nicoll, J.A.R. (2013). Cytokine gene polymorphisms and outcome after traumatic brain injury. J. Neurotrauma 30, 1710–1716.

167. Weisbrod, A.B., Rodriguez, C., Bell, R., Neal, C., Armonda, R., Dorlac, W., Schreiber, M., and Dunne, J.R. (2012). Long-term outcomes of combat casualties sustaining penetrating traumatic brain injury. J. Trauma Acute Care Surg. 73, 1525–1530.

168. Willmott, C., Ponsford, J., Downing, M., and Carty, M. (2014). Frequency and quality of return to study following traumatic brain injury. J. Head Trauma Rehabil. 29, 248–256.

169. Willmott, C., Withiel, T., Ponsford, J., and Burke, R. (2014). COMT Val158Met and cognitive and functional outcomes after traumatic brain injury. J. Neurotrauma 31, 1507–1514.

170. Wilson, L., Marsden-Loftus, I., Koskinen, S., Bakx, W., Bullinger, M., Formisano, R., Maas, A., Neugebauer, E., Powell, J., Sarajuuri, J., Sasse, N., Von Steinbuechel, N., Von Wild, K., and Truelle, J.-L. (2017). Interpreting quality of life after brain injury scores: Cross-Walk with the short form-36. J. Neurotrauma 34, 59–65.

171. Woischneck, D., Schmitz, B., and Kapapa, T. (2017). MRI detection of cerebral lesions in post-traumatic anisocoria: specificity and prognostic significance. Clin. Radiol. 72, 426.e7-426.e15.

172. Woischneck, D., Skalej, M., Firsching, R., and Kapapa, T. (2015). Decerebrate posturing following traumatic brain injury: MRI findings and their diagnostic value. Clin. Radiol. 70, 278–285.

173. Wright, D.W., Yeatts, S.D., Silbergleit, R., Palesch, Y.Y., Hertzberg, V.S., Frankel, M., Goldstein, F.C., Caveney, A.F., Howlett-Smith, H., Bengelink, E.M., Manley, G.T., Merck, L.H., Janis, L.S., Barsan, W.G., Investigators, N., and Wright DW Merck LH, Espinoza TR, Salomone JP, Dhall SS, Hudgins PA, Allen JW, Goldstein F, Hertzberg V, Rogers SD, Calcaterra AM, Howlett-Smith H, Lane B, Lunney MP, Cook N, Hall A, Hall A, McDougal A, Subramanian A, Pradilla G, Stein DG, Silbergleit R, F.M. (2014). Very early administration of progesterone for acute traumatic brain injury. N. Engl. J. Med. 371, 2457–2466.

174. Xu, G.-Z., Li, W., Liu, K.-G., Wu, W., Lu, W.-C., Zhang, J.-F., and Wang, M.-D. (2014). Early pressure dressing for the prevention of subdural effusion secondary to decompressive craniectomy in patients with severe traumatic brain injury. J. Craniofac. Surg. 25, 1836–1839.

175. Xu, L., Li, B., Yang, C., Li, C., and Peng, Y. (2016). Clinical research on postoperative efficacy and related factors of early simulation hyperbaric oxygen therapy for severe craniocerebral injury. Pak. J. Pharm. Sci. 29, 273–280.

176. Yang, C., Li, Q., Wu, C., Zan, X., and You, C. (2014). Surgical treatment of traumatic multiple intracranial hematomas. Neurosciences (Riyadh). 19, 306–311.

177. Yang, D.-B., Yu, W.-H., Dong, X.-Q., Zhang, Z.-Y., Du, Q., Zhu, Q., Che, Z.-H., Wang, H., Shen, Y.-F., and Jiang, L. (2017). Serum macrophage migration inhibitory factor concentrations correlate with prognosis of traumatic brain injury. Clin. Chim. Acta 469, 99–104.

178. Yao, S., Song, J., Li, S., Cao, C., Fang, L., Wang, C., and Xu, G. (2017). Helsinki Computed Tomography Scoring System Can Independently Predict Long-Term Outcome in Traumatic Brain Injury. World Neurosurg. 101, 528–533.

179. You, W., Feng, J., Tang, Q., Cao, J., Wang, L., Lei, J., Mao, Q., Gao, G., and Jiang, J. (2016). Intraventricular intracranial pressure monitoring improves the outcome of older adults with severe traumatic brain injury: an observational, prospective study. BMC Anesthesiol. 16, 35.

180. Yu, A.H., Cheng, C.H., Yeung, J.H.H., Poon, W.S., Ho, H. fai, Chang, A., and Rainer, T.H. (2012). Functional outcome after head injury: comparison of 12-45 year old male and female hormonally active patients. Injury 43, 603–607.

181. Yu, G.-F., Huang, Q., Dai, W.-M., Jie, Y.-Q., Fan, X.-F., Wu, A., Lv, Y., Li, Y.-P., and Yan, X.-J. (2012). Prognostic value of copeptin: one-year outcome in patients with traumatic brain injury. Peptides 33, 164–169.

182. Gaetani, P., Revay, M., Sciacca, S., Pessina, F., Aimar, E., Levi, D., and Morenghi, E. (2012). Traumatic brain injury in the elderly: considerations in a series of 103 patients older than 70. J. Neurosurg. Sci. 56, 231–237.

183. Yu, P., Tian, Q., Wen, X., Zhang, Z., and Jiang, R. (2015). Analysis of Long-Term Prognosis and Prognostic Predictors in Severe Brain Injury Patients Undergoing Decompressive Craniectomy and Standard Care. J. Craniofac. Surg. 26, e635-41.

184. Yu, W., Le, H.-W., Lu, Y.-G., Hu, J.-A., Yu, J.-B., Wang, M., and Shen, W. (2015). High levels of serum mannose-binding lectins are associated with the severity and clinical outcomes of severe traumatic brain injury. Clin. Chim. Acta. 451, 111–116.

185. Yuan, Q., Wu, X., Cheng, H., Yang, C., Wang, Y., Wang, E., Qiu, B., Fei, Z., Lan, Q., Wu, S., Jiang, Y., Feng, H., Liu, J., Liu, K., Zhang, F., Jiang, R., Zhang, J., Tu, Y., Wu, X., Zhou, L., and Hu, J. (2016). Is Intracranial Pressure Monitoring of Patients With Diffuse Traumatic Brain Injury Valuable? An Observational Multicenter Study. Neurosurgery 78, 361–369.

186. Yuan, Q., Wu, X., Yu, J., Sun, Y., Li, Z., Du, Z., Mao, Y., Zhou, L., and Hu, J. (2015). Effects and Clinical Characteristics of Intracranial Pressure Monitoring-Targeted Management for Subsets of Traumatic Brain Injury: An Observational Multicenter Study. Crit. Care Med. 43, 1405–1414.

187. Zador, Z., Sperrin, M., and King, A.T. (2016). Predictors of Outcome in Traumatic Brain Injury: New Insight Using Receiver Operating Curve Indices and Bayesian Network Analysis. PLoS One 11, e0158762.

188. Zafonte, R.D., Bagiella, E., Ansel, B.M., Novack, T.A., Friedewald, W.T., Hesdorffer, D.C., Timmons, S.D., Jallo, J., Eisenberg, H., Hart, T., Ricker, J.H., Diaz-Arrastia, R., Merchant, R.E., Temkin, N.R., Melton, S., and Dikmen, S.S. (2012). Effect of citicoline on functional and cognitive status among patients with traumatic brain injury: Citicoline Brain Injury Treatment Trial (COBRIT). JAMA 308, 1993–2000.

189. Zeng, J., Tong, W., and Zheng, P. (2013). Decreased risk of acute kidney injury with intracranial pressure monitoring in patients with moderate or severe brain injury. J. Neurosurg. 119, 1228–1232.

190. Zhang, L.-M., Li, R., Zhao, X.-C., Zhang, Q., and Luo, X.-L. (2017). Increased Transfusion of Fresh Frozen Plasma is Associated with Mortality or Worse Functional Outcomes After Severe Traumatic Brain Injury: A Retrospective Study. World Neurosurg. 104, 381–389.

191. Zhang, Z.-Y., Zhang, L.-X., Dong, X.-Q., Yu, W.-H., Du, Q., Yang, D.-B., Shen, Y.-F., Wang, H., Zhu, Q., Che, Z.-H., Liu, Q.-J., Jiang, L., and Du, Y.-F. (2014). Comparison of the performances of copeptin and multiple biomarkers in long-term prognosis of severe traumatic brain injury. Peptides 60, 13–17.

192. Zhao, L., Wang, W., Zhong, J., Li, Y.Y., Cheng, Y.Z., Su, Z., Zheng, W., and Guan, X.-D. (2016). The effects of magnesium sulfate therapy after severe diffuse axonal injury. Ther. Clin. Risk Manag. 12, 1481–1486.

193. Zhao, Y.-Y., Lou, L., Yang, K.-C., Wang, H.-B., Xu, Y., Lu, G., and He, H.-Y. (2017). Correlation of tenascin-C concentrations in serum with outcome of traumatic brain injury in humans. Clin. Chim. Acta 472, 46–50.

194. Zheng, P., He, B., Guo, Y., Zeng, J., and Tong, W. (2015). Decreased apparent diffusion coefficient in the pituitary and correlation with hypopituitarism in patients with traumatic brain injury. J. Neurosurg. 123, 75–80.

195. Walder, B., Haller, G., Rebetez, M., Delhumeau, C., Bottequin, E., Schoettker, P., Ravussin, P., Brodmann Maeder, M., Stover, J., Zürcher, M., Haller, A., Wäckelin, A., Haberthür, C., Fandino, J., Haller, C., and Osterwalder, J. (2013). Severe traumatic brain injury in a high-income country: an epidemiological study. J. Neurotrauma 30, 1934–1942.
